# How to Screen and Prevent Metabolic Syndrome in Patients of PCOS Early: Implications From Metabolomics

**DOI:** 10.3389/fendo.2021.659268

**Published:** 2021-06-02

**Authors:** Xiaoxuan Zhao, Xiaoling Feng, Xinjie Zhao, Yuepeng Jiang, Xianna Li, Jingyun Niu, Xiaoyu Meng, Jing Wu, Guowang Xu, Lihui Hou, Ying Wang

**Affiliations:** ^1^ Department of First Clinical Medical College, Heilongjiang University of Chinese Medicine, Harbin, China; ^2^ Department of Gynecology, the First Affiliated Hospital of Heilongjiang University of Chinese Medicine, Harbin, China; ^3^ Key Laboratory of Separation Science for Analytical Chemistry, Dalian Institute of Chemical Physics, Chinese Academy of Sciences, Dalian, China; ^4^ School of Basic Medical Sciences, Zhejiang Chinese Medical University, Hangzhou, China; ^5^ College of Pharmacy, Heilongjiang University of Chinese Medicine, Harbin, China; ^6^ Centre for Reproductive Medicine, The First Affiliated Hospital of Zhengzhou University, Zhengzhou, China

**Keywords:** metabolomics, polycystic ovary syndrome, metabolic syndrome, biomarkers, preventive medicine

## Abstract

**Background:**

Polycystic ovary syndrome (PCOS) is a complex reproductive endocrine disorder. And metabolic syndrome (MS) is an important bridge for PCOS patients to develop other diseases, such as diabetes and coronary heart disease. Our aim was to study the potential metabolic characteristics of PCOS-MS and identify sensitive biomarkers so as to provide targets for clinical screening, diagnosis, and treatment.

**Methods:**

In this study, 44 PCOS patients with MS, 34 PCOS patients without MS, and 32 healthy controls were studied. Plasma samples of subjects were tested by ultraperformance liquid chromatography (UPLC) system combined with LTQ-orbi-trap mass spectrometry. The changes of metabolic characteristics from PCOS to PCOS-MS were systematically analyzed. Correlations between differential metabolites and clinical characteristics of PCOS-MS were assessed. Differential metabolites with high correlation were further evaluated by the receiver operating characteristic (ROC) curve to identify their sensitivity as screening indicators.

**Results:**

There were significant differences in general characteristics, reproductive hormone, and metabolic parameters in the PCOS-MS group when compared with the PCOS group and healthy controls. We found 40 differential metabolites which were involved in 23 pathways when compared with the PCOS group. The metabolic network further reflected the metabolic environment, including the interaction between metabolic pathways, modules, enzymes, reactions, and metabolites. In the correlation analysis, there were 11 differential metabolites whose correlation coefficient with clinical parameters was greater than 0.4, which were expected to be taken as biomarkers for clinical diagnosis. Besides, these 11 differential metabolites were assessed by ROC, and the areas under curve (AUCs) were all greater than 0.7, with a good sensitivity. Furthermore, combinational metabolic biomarkers, such as glutamic acid + leucine + phenylalanine and carnitine C 4: 0 + carnitine C18:1 + carnitine C5:0 were expected to be sensitive combinational biomarkers in clinical practice.

**Conclusion:**

Our study provides a new insight to understand the pathogenesis mechanism, and the discriminating metabolites may help screen high-risk of MS in patients with PCOS and provide sensitive biomarkers for clinical diagnosis.

## Background

Polycystic ovary syndrome (PCOS) is a common endocrine-metabolic disorder that affects 12–18% women, depending on the diagnostic criteria used (1). Its main clinical manifestations include hirsutism, hypoovulation, and polycystic ovary morphology, *etc.* (2). Patients with PCOS are at high risk for metabolic diseases. Bhattacharya confirmed that Indian girls with PCOS were 4.2 times more likely to develop metabolic syndrome (MS) than girls without PCOS (3). Moreover, a meta-analysis of 107 studies by Jamal Hallajzadeh et al. showed a significant relationship between PCOS and MS (4). Therefore, PCOS should no longer be considered as a simple gynecological disease. Besides, women with MS show an increased long-term risk of type 2 diabetes (T2DM), cardiovascular disease (CVD), and certain types of cancers (5). Thus, longitudinal, in-depth and dynamic understanding of abnormal metabolic characteristics from health to PCOS, even to MS is desperately needed to early identify disorders so as to avoid poor outcomes. Current clinical indicators, such as body mass index (BMI), fasting insulin (FINS), and high density lipoprotein (HDL), while useful in determining MS risk and representative established diseases, contribute little to our understanding of disease pathology. Therefore, screening sensitive biomarkers in patients of PCOS that tend to develop MS is urgent, so as to achieve better prediction and prevention effects and effectively avoid huge health care costs.

**Figure 1:**
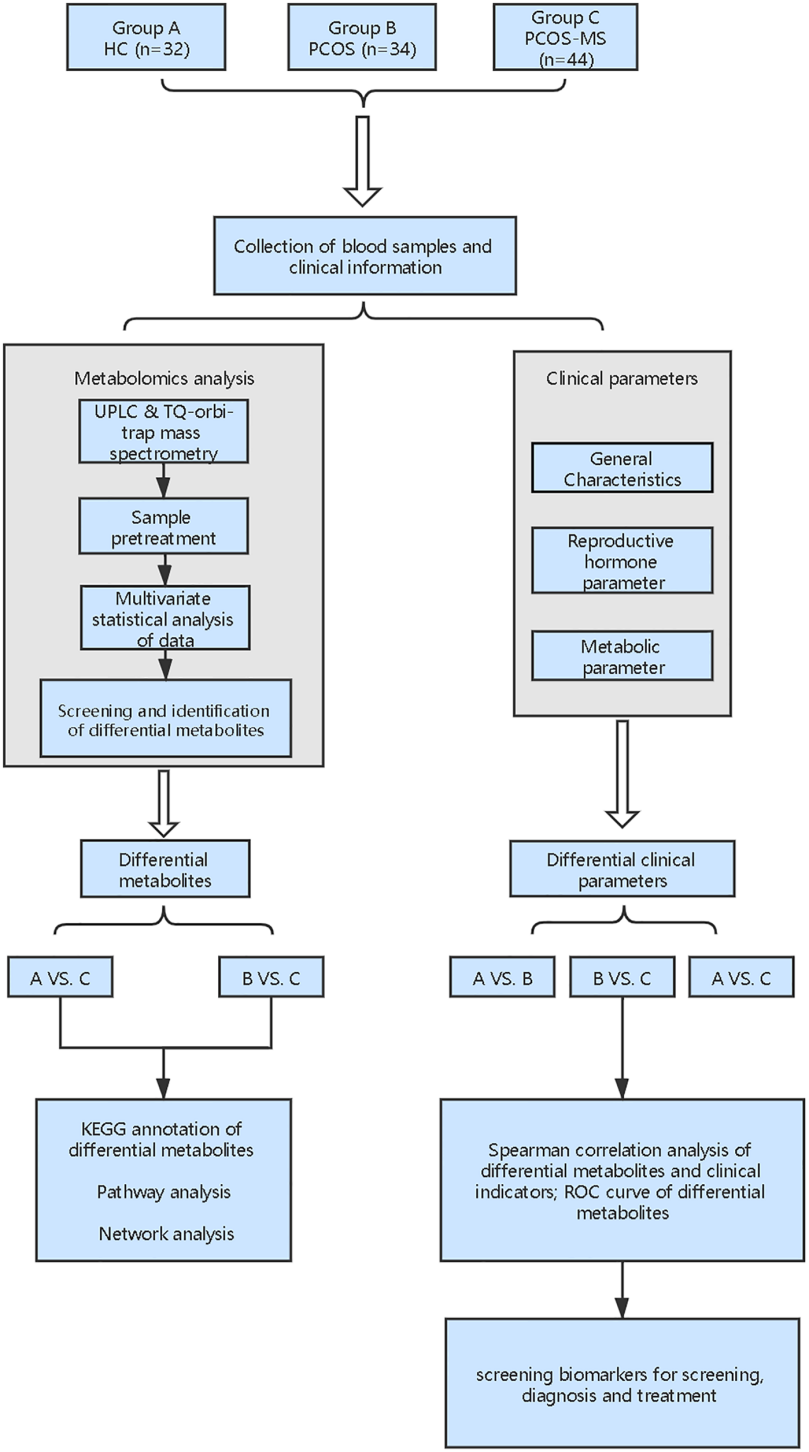
Flowchart of study strategy in this study.

Metabonomics mainly studies the small molecule metabolites at the end of the bioinformatics chain, which can sensitively reflect authentic biological activities in the organism and is closely related to traditional biological and clinical endpoints (6). From a clinical perspective, the implementation of metabonomics will facilitate the development of predictive and personalized models that fully consider the heterogeneity of treatment response and disease stratification. Therefore it is a novel and useful tool in pharmacology and personalized diagnosis (7). Currently, the current metabolomic studies on PCOS mainly focus on the differences between PCOS and healthy control (HC) women or concern the differences between different phenotypes of PCOS, such as hyperandrogen and non-hyperandrogen, fat and thin, *etc.*, but there is still a lack of metabonomics studies to longitudinally explore the development of PCOS to MS. Thus, in this study, we recruited three types of volunteers including HC women, patients with PCOS, or PCOS-MS and focused on analysis of metabolic characteristics between PCOS and PCOS-MS. Besides, we explored the relationship between differential metabolites and clinical characterization in these two groups to identify biomarkers for the prediction, diagnosis, and target-treatment of disease. The flowchart of the study strategy is shown in **Figure 1**. Our experiment makes up for the gap of current researches and provides directions for the prevention and truncation of disease course.

## Methods

### Subjects

In this study 44 PCOS patients with MS (PCOS-MS group), 34 PCOS patients without MS risk factors (PCOS group), and 32 age-matched healthy women (HC group) were enrolled. They all visited the gynecological outpatient department or physical examination center of the First Affiliated Hospital of Heilongjiang University of Chinese Medicine from March 2018 to March 2020. All the subjects were between the ages of 18 and 35 and had menstrual history for at least two years. The diagnosis of PCOS should conform to the Rotterdam criteria of 2003 (8). The diagnosis of MS must be in accordance with the third guidelines for the adult treatment group of the national cholesterol education program (NCEP ATP III) (9): (1) central obesity: waist circumference (WC) ≥80 cm; (2) triglyceride (TG) >1.7 mmol/L; (3) HDL <1.3 mmol/L; (4) systolic blood pressure (SBP) ≥130 mmHg or diastolic blood pressure (DBP) ≥85 mmHg, or having been diagnosed with hypertension; (5) fasting blood glucose (FBG) ≥5.6 mmol/L, or having been diagnosed with diabetes. The HC group had regular menstrual cycles (27–35 days) without clinical or biochemical manifestations of hyperandrogen. Besides, women who had adrenal, thyroid, and pituitary dysfunction or who used medications that interfered with endocrine, blood pressure, lipid, or carbohydrate metabolism during the first six months, such as oral contraceptives, androgen preparations, insulin sensitizers, iron supplements were excluded from the study. This study complied with the Declaration of Helsinki in clinical research and was approved by the Ethics Committee of Heilongjiang University of Traditional Chinese Medicine (NO. HZYLLKT201500401). Besides, informed consent was obtained from all participants.

### Collection of Serum Samples and Clinical Data

After three days of 300 g carbohydrate diet and 12 h of fasting at night, 20 ml of fasting blood samples was collected at 08:00–09:00 a. m. on the 2nd–4th days of natural menstrual cycle or withdrawal bleeding by taking 10 mg of oral dydrogesterone for 7 days in a row. The samples were placed at room temperature for 30 min, centrifuged at 3,000 r/min for 10 min, and then 8 ml of serum was separated and subpacked in 1.5 ml EP tubes. Part of the serum was stored at −80°C for metabonomics test. The other part was sent to the biochemistry and isotope departments of the First Affiliated Hospital of Heilongjiang University of Traditional Chinese Medicine for reproductive hormone tests and biochemical examination. The specific test items were as follows. Reproductive hormone was determined by chemiluminescence including blood follicle stimulating hormone (FSH), luteinizing hormone (LH), total testosterone (TT), dehydroepiandrosterone sulfate (DHEAS), androstenedione (A2), sex hormone binding globulin (SHBG). Glucose metabolism indicators were measured by chemiluminescence including FBG, FINS, homeostatic model index of insulin resistance (HOMA-IR), HOMA-*β*. Lipid metabolism was assessed by chemiluminescence, including total cholesterol (TC), TG, low density lipoprotein cholesterol (LDL-C), HDL-C, apolipoprotein A 1 (ApoA1), apolipoprotein B (ApoB). The calculation formula was shown as follows: HOMA-IR = FBG (mmol/L) × FINS (μIU/ml)/22.5 (10), free androgen index (FAI) = TT (nmol/L) × 100/SHBG (nmol/L), atherosclerotic index (AI) = TC-HDL-C/HDL-C (11).

After the blood samples are collected, the subjects fasted for physical examination, and the relevant data were recorded, including SBP, DBP, height, weight, WC, hip circumference, Ferriman–Gallwey (F–G) score. BMI and waist to hip ratio (WHR) were calculated.

### Serum Metabolomic Analysis

Metabolomic analysis was performed in the Key Laboratory of Separation Science for Analytical Chemistry of Dalian Institute of Chemical Physics, and the specific operation process was as follows: serum thawed at 4°C and 400 µl acetonitrile containing 11 internal standards were added to every 100 µl serum samples to deproteinize. The 11 internal standards were choline-d4, carnitinen C2: 0-d3, carnitine C10: 0-d3, carnitine C16: 0-d3, cholic acid-d4, chenodeoxycholic acid-d4, LPC 19:0, phenylalanie-d5, tryptophand5, Palmitic acid-d3, and stearic acid-d3 (Sigma–Aldrich, St. Louis, MO, USA). After oscillation, the supernatant was separated by centrifugation at 14,000 g centrifugal force for 12 min at 4°C. The supernatant was evenly divided into two parts for freeze-drying and stored in a refrigerator at −80°C. Before injection, the sample was redissolved in acetonitrile−water by 1:4 volume. After the oscillation, the samples were centrifuged at 14,000g centrifugal force for 10 min at 4°C, and the supernatant was taken for sample injection. Then 3 μl aliquot of each sample was injected onto the column in positive ion mode and 4 μl in negative ion mode.

Ultraperformance liquid chromatography (UPLC) system (Waters Corporation Milford, MA, USA) combined with LTQ-orbi-trap mass spectrometry (Thermo Fisher Scientific, Waltham, MA, USA) was applied to analyze the metabolic profiling in both electrospray ionization (ESI) positive and negative ion modes. For the ESI+ mode, chromatographic separation was performed on a Waters ACQUITY C18 (100 × 2.1 mm, 1.7 µm) (Waters, Ireland), and the mobile phase was contained in A (H2O/HCOOH = 100:1) and B (CH3CN/HCOOH = 100:1) with the flow rate of 0. 35 ml/min. The gradient program was as follows: 0–1 min 5% B; 1–15 min 5–100% B; 15–18 min 100%; 18–18.5 min 100–5% B; 18.5–2.0 min 5% B. For the ESI-negative mode, chromatographic separation was performed on a Waters ACQUITYTM T3 (2.1 × 100 mm, 1.8 μm) (Waters, Ireland), and the mobile phase was contained in A (6.5 mmol/L ammonium bicarbonate water solution) and B (6.5 mmol/L ammonium bicarbonate in 98% methanol and water) (95:5, v/v.) The gradient program was 2% A for 1 min, changed to 100% B linearly within 18 min and held for 3 min, and finally back to 2% B. Flow rate was 0.35 ml/min, and the column temperature was kept at 50°C both in positive ion mode and negative ion mode.

The mass spectrometry detection settings either in the positive or negative ion mode were as follows: mass spectrometry scan with mass range of m/z 80–1,000 Da, with a desolvation gas temperature of 350°C, drying gas flow rate at 11 L/min nitrogen, capillary voltage at 4.0 kV, and fragmentor voltage at 230 V.

### Data Processing and Multivariate Analysis

SIEVE 1.2 version Workstation (Thermo Fisher Scientific, Waltham, MA, USA) was utilized to identify and match the peaks of the original data. After removing zero value with 80% rule (12), the intensity of each reserved peak was corrected with an internal standard to reduce the system error. SIMCA-P 11.0 version (Umetrics, Umea, Sweden) was used for multivariate statistical analysis where the data were preprocessed by unit variance (UV) scaling and mean centering before performing principal component analysis (PCA) and orthogonal partial least squares discriminant analysis (OPLS-DA). Variable importance in the projection (VIP) value was used to select the target metabolites which significantly changed between compared groups, with the standard of VIP >5 and *P <*0. 05. The target metabolites were determined by mass spectrometric technology. Internal verification of seven-fold cross validation and response permutation test were adopted to evaluate the prediction ability of the model. All the results of the metabolomic analysis were computed by IBM SPSS software (25. 0 version, SPSS Inc., USA).

### Statistical Analysis

Statistical analysis was performed by using SPSS 25.0 (SPSS, Chicago, IL, USA) and SAS 9.3(SAS Institute Inc., Cary, NC, USA). If the data were normally distributed and homogenous, ANOVA was used to compare the clinical indicators of HC, PCOS, and PCOS-MS groups. Data were described by mean ± standard deviation (SD). For non-homogeneous data, we used Kruskal–Wallis tests, and the result should be described by median with 25–75% interquartile range. Spearman correlation analysis was conducted to analyze the correlation between different metabolites and clinical indicators. The differences were considered statistically significant if P <0.05.

## Results

### Comparison of Clinical Features and Biochemical Indicators

The general characteristics, reproductive hormone, and metabolic parameters of the subjects were summarized in **Table 1**. We found that only F–G score, reproductive endocrine hormone (FSH, LH, LH/FSH, TT, A2), and LDL-C were significantly changed in the PCOS group when compared with the HC group (*P <* 0.05). It was obvious that the changes of hypothalamus–pituitary–ovarian (H–P–O) axis rather than glucolipid metabolism were remarkable in PCOS patients. Moreover, even excluding the diagnosis of MS, the level of LDL-C in the PCOS group was still significantly higher than that in the HC group. LDL-C is a lipoprotein particle that carries cholesterol into peripheral tissue cells and can be converted into oxidative modification of LDL (OX-LDL). When LDL, especially the OX-LDL is too much, the level of cholesterol can be accumulated in the arterial walls, leading to an increased risk of CVD (13). Our study was consistent with the research of Zhao et al. (14). It is suggested that clinicians should also pay attention to screening lipid metabolism indicators in the diagnosis of lean PCOS patients so as to early detect the susceptibility of metabolism disorders and take prevention measures against the development of the disease. It was interesting that we did not find IR in subjects of the PCOS group, which also suggested that though IR was very common in PCOS (15), it was not universal, reflecting the metabolic heterogeneity of PCOS (15). Of course, these patients may develop IR in the future as the course of the disease progresses, but we could discover that abnormalities in lipid metabolism were initiated before the disorder of glucose metabolism occurred. Therefore, the mechanism between abnormal lipid metabolism and reproductive hormones in PCOS still deserves our in-depth attention.

**Table 1:** General characteristics, reproductive hormone and metabolic parameters among groups of HC, PCOS and PCOS-MS.

Clinical features	HC group (n = 32)	PCOS group (n = 34)	PCOS-MS (n = 44)	adjusted *P* value HC *vs*. PCOS	Adjusted *P* value HC *vs*. PCOS-MS	Adjusted *P* value PCOS *vs*. PCOS-MS
**General Characteristics**						
Age (years)	25.84 ± 2.71	25.06 ± 3.25	26.30 ± 4.51	0.303	0.616	0.181
Menarche age (years)	13.50 ± 1.19	14.09 ± 1.74	13.02 ± 1.30^#^	0.132	0.107	0.003
SBP (mmHg)	101.66 ± 9.73	104.97 ± 9.55	111.14 ± 13.29*^#^	0.262	0.001	0.025
DBP (mmHg)	70.94 ± 7.72	72.06 ± 6.30	76.97 ± 10.62*^#^	0.657	0.008	0.019
BMI (kg/m^2^)	19.50 ± 1.33	19.61 ± 1.64	28.99 ± 3.04*^#^	0.820	0.000	0.000
WC(cm)	72.47 ± 3.78	72.97 ± 4.79	95.46 ± 7.22*^#^	0.634	0.000	0.000
WHR	0.80 ± 0.03	0.81 ± 0.03	0.92 ± 0.04*^#^	0.142	0.000	0.000
F-G score	0(0–1)	2.5(2–5.25)*	4(3.25–5.75)*^#^	0.000	0.000	0.019
**Reproductive hormone parameter**						
FSH (mIU/ml)	6.94 ± 2.30	4.48 ± 1.03*	4.17 ± 1.28*	0.000	0.000	0.247
LH (mIU/ml)	5.82(3.92–6.79)	8.05(4.63–16.39)*	6.63(4.23–10.43)	0.024	0.185	0.190
LH/FSH	0.93(0.63–1.02)	1.53(1.07–3.63)*	1.80(0.92–2.59)*	0.000	0.000	0.379
Testosterone (ng/dl)	28.03 ± 13.07	43.59 ± 22.08*	58.79 ± 26.28*^#^	0.001	0.000	0.008
DHEAS(ug/dl)	192.26 ± 73.41	238.57 ± 99.96	217.08 ± 115.41	0.063	0.353	0.390
A2(ng/ml)	1.90(1.60–2.58)	5.76(4.06–8.32)*	5.56(3.84–15.0)*	0.000	0.000	0.486
SHBG (nmol/l)	50.49 ± 31.38	49.82 ± 26.80	18.61 ± 10.32*^#^	0.902	0.000	0.000
FAI	0.91(0.50–1.70)	1.35(0.76–2.30)	5.30(3.11–8.05)*^#^	0.128	0.000	0.000
**Metabolic parameter**						
FBG(mmol/l)	4.62 ± 0.33	4.74 ± 0.34	5.60 ± 1.12*^#^	0.116	0.000	0.000
Fasting insulin (μIU/ml)	7.68 ± 3.21	9.26 ± 3.74	24.14 ± 11.38*^#^	0.077	0.000	0.000
HOMA-IR	1.59 ± 0.69	1.96 ± 0.85	6.05 ± 3.09*^#^	0.064	0.000	0.000
HOMA-β	136.73(100.61–171.62)	134.15(100.38–190.71)	245.02(143.47–349.18)*^#^	0.617	0.000	0.000
TC(mmol/l)	3.86 ± 0.54	4.08 ± 0.54	4.98 ± 0.86*^#^	0.211	0.000	0.000
LDL-C (mmol/L)	2.05 ± 0.46	2.57 ± 0.61*	3.16 ± 0.79*^#^	0.005	0.000	0.000
TG (mmol/l)	0.81(0.63–0.96)	0.85(0.57–1.13)	2.12(1.76–3.13)*^#^	0.174	0.000	0.000
HDL-C (mmol/l)	1.39 ± 0.10	1.44 ± 0.12*	1.08 ± 0.17*^#^	0.005	0.000	0.000
AI	1.79 ± 0.35	1.83 ± 0.45	3.67 ± 0.88*^#^	0.777	0.000	0.000
TG/HDL-C	0.56(0.47–0.72)	0.58(0.37–0.79)	2.05(1.52–2.96)*^#^	0.817	0.000	0.000
Apolipoprotein B/A1	0.53 ± 0.12	0.53 ± 0.13	0.87 ± 0.21*^#^	0.786	0.000	0.000

*represents P < 0.05 when the other two groups compared with HC group; ^#^represents P < 0.05 when the other two groups compared with PCOS-MS group.

There were differences in general characteristics (except for age menarche age), reproductive hormone (except for LH and DHEAS), and metabolic parameters between groups of PCOS-MS and HC. Besides, LH did not increase as much in the PCOS-MS group as in the PCOS group, and studies showed that LH tended to be more normal, not worse, in obese women with PCOS (16). The study showed that the LH pulse amplitude was the highest in lean PCOS, while it was relatively normal in the overweight PCOS patients, whose serum LH tended to increase when they lost weight for more than 3 to 6 months, which indicated that some metabolic factors related to weight might be the driving factors for the change of LH secretion (17). In this table, we found that there was no significant increase in DHEAS, an adrenal precursor androgen marker, between groups of HC and PCOS whether with MS or not. Data showed that only about 20–30% of patients with PCOS showed excessive secretion of adrenal precursor androgen (18). It indicated that elevated level of androgen levels in PCOS, especially in the PCOS-MS group was mainly due to the abnormality of the hypothalamic–pituitary–ovarian axis, which matched with the result that TT and A2 of PCOS-MS were respectively two and four times of those in the HC group.

In addition to some differential MS-related indicators between PCOS and PCOS-MS, we also detected some imperceptible indicators that could reflect the differences in endocrine characteristics between the two groups, including menarche age, TT, FAI, *etc.* We found that the menarche age in the PCOS-MS group was earlier than that in the PCOS group. Studies found that menarche age was affected by various factors, including heredity, diet habits, natural environment, *etc.* We analyzed that patients in the PCOS-MS group were more accessible to various fat source at an early age due to the influence of heredity or diet habits, which may be a vital information for the secretion of leptin, finally stimulating the hypothalamus and leading to the oversecretion of GnRH. In addition, GnRH stimulates the pituitary–ovarian axis and initiates the acceleration of puberty (19). A study by Kazem Mohamad et al. including 488 girls between 11 and 17 years in southern Iran showed that higher BMI was associated with lower menarche age (20). This particular genetic and dietary factor may also underlie their later development into MS. Besides, TT and FAI were higher in PCOS-MS than in PCOS, we speculated that the abnormal metabolism of reproductive hormone would be aggravated after the onset of MS. Studies have shown that obesity can lead to changes in endocrine and/or metabolic patterns, hormone transport and/or its interaction with the target tissue, resulting in an abnormal concentration of androgen in the peripheral blood (21). In **Table 1**, each glycolipid metabolism indicator in the group PCOS-MS was different from that in the group PCOS, which may be related to the increase of androgen level. Moreover, studies have shown that the increase of androgen and decrease of SHBG may contribute to the development of MS and T2DM (22). Hyperactive androgen is the main reason for the increase of visceral adipose tissue (23). Thus, it can be concluded that glycolipid metabolism and reproductive hormones interact to promote the development of the disease, which is consistent with our results. For example, in our investigation, the increased level of indicators related to glycolipid metabolism was accompanied by the increase of TT and FAI in the PCOS-MS group. In addition, we also found an interesting phenomenon that the decreased level of LH in PCOS-MS was contrary to the increased trend of TT when compared with the PCOS group. Our result was consistent with the research studies by Mu et al. (24) and Li et al. (25) who considered that there was no significant difference in DHEAS, an adrenal gland-derived androgen, between the two groups; we speculated that the increased androgen may be due to the following aspects: 1). the increased level of follicular atresia in PCOS-MS patients due to chronic inflammation leads to the decrease of aromatase activity in the body and the increase of testosterone. 2). The enzyme system required for the synthesis of various ovarian steroids is dysregulated in PCOS patients, especially in PCOS-MS (26). For example, the active function of 17-hydroxysteroid dehydrogenase leads to the increased conversion of androsterone to testosterone. 3). Since 50% of testosterone comes from the transformation of peripheral tissues and adipose tissue, skin and liver are important sites involved in peripheral transformation; the increase of adipose tissue and abnormal liver metabolism in patients of PCOS-MS may be important sources of testosterone (27). Specific intracellular mechanisms in female allow each cell to control androgen availability according to its own needs, regardless of the influence of the rest of the body. This mechanism is completely different from the male endocrinology, and this highly complex mechanism needs further exploration.

### Analysis of the Serum Metabolic Profiling by UPLC LTQ-Orbi-trap Mass Spectrometry

#### Pattern Recognition of the Comparisons of PCOS *vs*. HC, PCOS *vs*. PCOS-MS and PCOS-MS *vs*. HC

**Figure 2:**
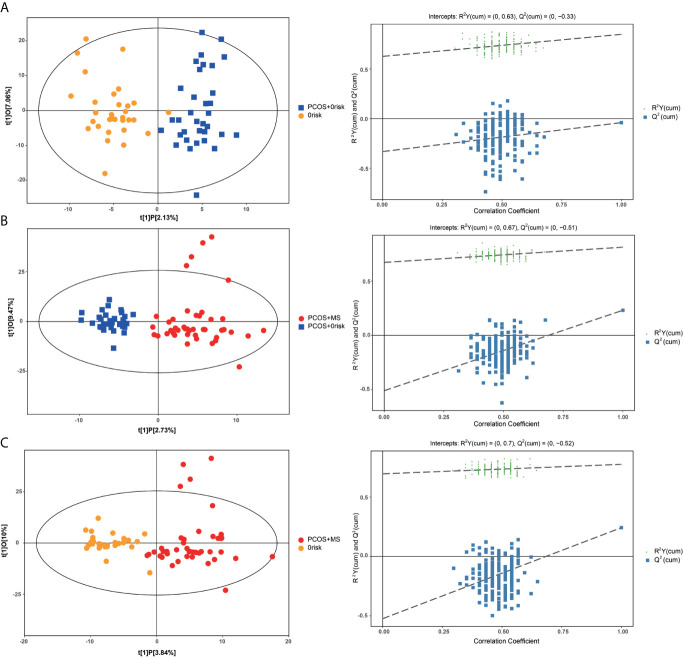
OPLS-DA cross-validated scores (left column) and the permutation test (right column) of UPLC combined with LTQ-orbi-trap mass spectrometry of plasma samples from HC, PCOS, PCOS-MS. **(A)** PCOS *vs*. HC; **(B)** PCOS-MS *vs*. PCOS; **(C)** PCOS-MS *vs*. HC.

A series of multivariate variable pattern recognition analysis were carried out. Firstly, PCA was performed to determine the distributions of and separations between different groups, and the results were shown in **Supplement Figure 1**. In the PCA score plot, we found a separation *via* pairwise contrasts, indicating metabolic differences in metabolic status between groups. To maximize the distinction, the OPLS-DA model was applied as shown in **Figure 2**. The result showed a significant difference in PCOS-MS whether compared to the HC or PCOS group. However, there was no significant difference between PCOS and HC after further univariate analysis. This suggested that the occurrence of PCOS was mainly due to the abnormality of the hypothalamic–pituitary–ovarian axis rather than metabolism changes. Besides, the permutation test was carried out to confirm the robustness of the model and the reliability of the results. As we could see in **Figure 2**, the models established were in accord with the real situation without over-fitting phenomenon. Therefore, we focus on the analysis of metabolic differences in PCOS-MS when compared to PCOS or HC groups, especially the former comparison, so as to explore the metabolic changes from PCOS to PCOS-MS. Such research helps us predict the development of diseases and find out sensitivity screening indicators for screening high-risk population for MS in patients with PCOS at early stage and cutting off the development of the disease course.

#### Analysis of Differential Metabolites in Groups of PCOS *vs*. PCOS-MS and PCOS-MS *vs*. HC

**Figure 3:**
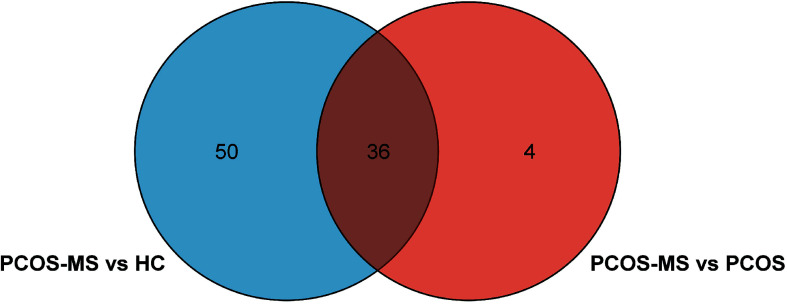
Venn diagram of differential metabolites in groups of HC, PCOS, PCOS-MS.

First, differential metabolites were obtained, and then various databases were searched for further analysis and identification, including METLIN (https://metlin.scripps.edu/), Kyoto Encyclopedia of Genes and Genomes (KEGG) (http://www.kegg.jp/kegg/pathway.html), Human Metabolome Database (HMDB) (http://www.hmdb.ca/), and PubChem Database (https://pubchem.ncbi.nlm.nih.gov/). The results were shown in **Tables 2**, **3**. In addition, after sorting out the substance names and classification, the Venn diagram was obtained by TB tools as shown in **Figure 3**.

**Table 2:** Differential metabolites between PCOS group and PCOS-MS group.

Metabolite	PCOS-MS and PCOS	Class	Pathway
FFA 16:0	↑*	free fatty acid	lipid metabolism
FFA 16:2	↑*	free fatty acid	lipid metabolism
FFA 16:3	↑*	free fatty acid	lipid metabolism
FFA 17:1	↑*	free fatty acid	lipid metabolism
FFA 20:3	↑*	free fatty acid	lipid metabolism
FFA 20:5-1	↑*	free fatty acid	lipid metabolism
FFA 22:5	↑*	free fatty acid	lipid metabolism
FFA 22:6	↑*	free fatty acid	lipid metabolism
Aliphatic amine C16:0	↑*	free fatty acid	lipid metabolism
Carnitine C4:0	↑*	carnitine	fatty acid β oxidation
Carnitine C5:0	↑*	carnitine	fatty acid β oxidation
Carnitine C18:1	↑*	carnitine	fatty acid β oxidation
LPC 14:0	↑*	lysophosphatidyl choline	phospholipid metabolism
LPC 16:0	↑*	lysophosphatidyl choline	phospholipid metabolism
LPC 16:1	↑*	lysophosphatidyl choline	phospholipid metabolism
LPC 20:3	↑*	lysophosphatidyl choline	phospholipid metabolism
LPC 22:4	↑*	lysophosphatidyl choline	phospholipid metabolism
LPC 22:5	↑*	lysophosphatidyl choline	phospholipid metabolism
glutamic acid	↑*	amino acid	amino acid metabolism
N -acetyl-L-lysine	↑*	amino acid	amino acid metabolism
*γ*-glut-leucine	↑*	amino acid	amino acid metabolism
Phenylalanine–Phenylalanine	↑*	amino acid	amino acid metabolism
gly-phe	↑*	amino acid	amino acid metabolism
L-kynurenine	↑*	amino acid	amino acid metabolism
Leucine	↑*	amino acid	amino acid metabolism
phenylalanine	↑*	amino acid	amino acid metabolism
Ornithine	↑*	amino acid	amino acid metabolism
tryptophane	↑*	amino acid	amino acid metabolism
valine	↑*	amino acid	amino acid metabolism
tyrosine	↑*	amino acid	amino acid metabolism
Glutamic acid	↑*	amino acid	amino acid metabolism
Uric Acid	↑*	Purine end products	purine metabolism
Hippuric acid	↓*	purine derivative	purine metabolism
GUDCA	↑*	ursodexycholic acid	bile acid metabolism
lactamide	↑*	lactamide	Glycolysis metabolism
2-Aminoethylphosphonic acid	↓*	NA	NA
Asp-Phe	↑*	NA	NA
D-Mannitol	↑*	NA	NA
N-Acetylneuraminic acid	↑*	NA	NA
Propylparaben	↑*	NA	NA

*Represents p < 0.05 when compared PCOS-MS with PCOS.

**Table 3:** Differential metabolites between HC group and PCOS-MS group.

Metabolite	PCOS-MS and HC	Class	Pathway
carnitine	↑*	carnitine	fatty acid *β* oxidation
carnitine 4:0	↑*	carnitine	fatty acid *β* oxidation
carnitine C14:1	↑*	carnitine	fatty acid *β* oxidation
carnitine C18:1	↑*	carnitine	fatty acid *β* oxidation
carnitine C18:2	↑*	carnitine	fatty acid *β* oxidation
carnitine C2:0	↑*	carnitine	fatty acid *β* oxidation
carnitine C5:0	↑*	carnitine	fatty acid *β* oxidation
carnitine C6:0	↑*	carnitine	fatty acid *β* oxidation
FFA 14:0	↑*	free fatty acid	lipid metabolism
FFA 15:0	↑*	free fatty acid	lipid metabolism
FFA 16:0	↑*	free fatty acid	lipid metabolism
FFA 16:1	↑*	free fatty acid	lipid metabolism
FFA 16:2	↑*	free fatty acid	lipid metabolism
FFA 16:3	↑*	free fatty acid	lipid metabolism
FFA 17:0	↑*	free fatty acid	lipid metabolism
FFA 17:1	↑*	free fatty acid	lipid metabolism
FFA 18:1	↑*	free fatty acid	lipid metabolism
FFA 18:2	↑*	free fatty acid	lipid metabolism
FFA 18:3	↑*	free fatty acid	lipid metabolism
FFA 18:4	↑*	free fatty acid	lipid metabolism
FFA 19:1	↑*	free fatty acid	lipid metabolism
FFA 20:1	↑*	free fatty acid	lipid metabolism
FFA 20:2	↑*	free fatty acid	lipid metabolism
FFA 20:3	↑*	free fatty acid	lipid metabolism
FFA 20:4	↑*	free fatty acid	lipid metabolism
FFA 20:5-1	↑*	free fatty acid	lipid metabolism
FFA 20:5-2	↑*	free fatty acid	lipid metabolism
FFA 22:2	↑*	free fatty acid	lipid metabolism
FFA 22:5	↑*	free fatty acid	lipid metabolism
FFA 22:6	↑*	free fatty acid	lipid metabolism
FFA 24:1	↑*	free fatty acid	lipid metabolism
LPC 14:0	↑*	lysophosphatidyl choline	phospholipid metabolism
LPC 15:0	↑*	lysophosphatidyl choline	phospholipid metabolism
LPC 16:0	↑*	lysophosphatidyl choline	phospholipid metabolism
LPC 16:1	↑*	lysophosphatidyl choline	phospholipid metabolism
LPC 18:0	↑*	lysophosphatidyl choline	phospholipid metabolism
LPC 20:3	↑*	lysophosphatidyl choline	phospholipid metabolism
LPC O-16:0	↑*	lysophosphatidyl choline	phospholipid metabolism
LPC18:0	↑*	lysophosphatidyl choline	phospholipid metabolism
PC 30:0	↑*	lysophosphatidyl choline	phospholipid metabolism
PC 34:1	↑*	lysophosphatidyl choline	phospholipid metabolism
PC 34:2	↑*	lysophosphatidyl choline	phospholipid metabolism
PC 36:5	↑*	lysophosphatidyl choline	phospholipid metabolism
PC 38:5	↑*	lysophosphatidyl choline	phospholipid metabolism
SM 34:1	↑*	Sphingo myexin	phospholipid metabolism
SM 36:2	↑*	Sphingo myexin	phospholipid metabolism
SM(d18:1/14:0)	↑*	Sphingo myexin	phospholipid metabolism
SPHINGOSINE-1-PHOSPHATE	↑*	Sphingosine phosphate	NA
Asp-Phe	↑*	Aspartate - phenylalanine	amino acid metabolism
DL-Proline	↑*	proline	amino acid metabolism
Glutamic acid	↑*	glutamic acid	amino acid metabolism
gly-phe	↑*	alanine	amino acid metabolism
leucine	↑*	leucine	amino acid metabolism
L-Isoleucine	↑*	isoleucine	amino acid metabolism
L-Lysine	↑*	lysine	amino acid metabolism
Ornithine	↑*	ornithine	amino acid metabolism
Phenylalanine	↑*	phenylalanine	amino acid metabolism
Tryptophan	↑*	tryptophane	amino acid metabolism
Tyrosine	↑*	tyrosine	amino acid metabolism
Valine	↑*	valine	amino acid metabolism
bilirubin	↑*	bilirubin	bile acid metabolism
GUDCA	↑*	ursodexycholic acid	bile acid metabolism
GUDCS	↑*	Oxycholic acid salt	bile acid metabolism
glycodeoxycholate	↓*	glycodeoxycholate	bile acid metabolism
uric acid	↑*	uric acid	purine metabolism
13,14-DIHYDRO-15-KETO PROSTAGLANDIN A2	↑*	NA	NA
13-cis-acitretin	↑*	NA	NA
3-Indolepropionic acid	↓*	NA	NA
5a-Dihydrotestosterone sulfate	↑*	NA	NA
Androsterone sulfate	↑*	NA	NA
D-Mannitol	↑*	NA	NA
Erucamide	↓*	NA	NA
Etiocholanolone sulfate	↑*	NA	NA
Hippuric acid	↓*	NA	NA
Indolelactic acid	↑*	NA	NA
lactamide	↑*	NA	NA
L-Altrose	↑*	NA	NA
L-kynurenine	↑*	NA	NA
LPE 22:6	↑*	NA	NA
N-Acetylneuraminic acid	↑*	NA	NA
N*α*-Acetyl-L-Iysine	↑*	NA	NA
phenylacetylglutamine	↓*	NA	NA
phe-phe	↑*	NA	NA
Propylparaben	↑*	NA	NA
p-xylene	↓*	NA	NA
*γ*-Glu-Leu	↑*	NA	NA

*Represents p < 0.05 when compared PCOS-MS with HC.

#### Differential Metabolites Between Groups of PCOS and PCOS-MS

**Figure 4:**
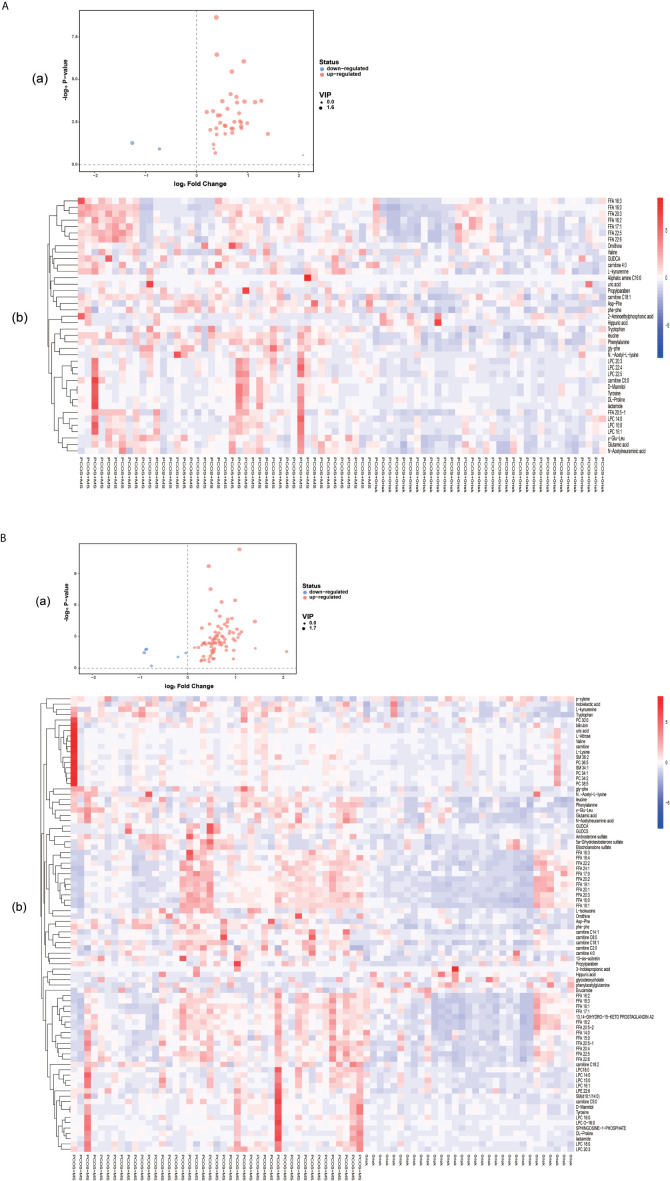
**(A)** Volcano plot (a) and heatmap of hierarchical clustering analysis (b) for group PCOS *vs*. PCOS-MS. **(B)** Volcano plot (a) and heatmap of hierarchical clustering analysis (b) for group HC *vs.* PCOS-MS The Volcano map shows differential metabolites in PCOS *vs.* PCOS-MS and HC *vs*. PCOS-MS, with the abscissa representing log2 (fold change) and the ordinate representing −log10 (P value). Metabolites which changed significantly (VIP > 1, P < 0.05) are highlighted in red (for increased) and blue (for decreased). Heatmaps can visually show the variation of differential metabolites *via* pairwise contrasts. Each small square represents a metabolite, and the color indicates the amount of metabolite, with the higher the amount, the darker the color (red is the high expression, blue is the low expression). The tree graph on the left shows the results of cluster analysis of the metabolite variation trend in the two groups of samples.

A total of 40 differential metabolites were obtained as shown in **Table 2**. The results were visualized in volcano plot as shown in **Figures 4A(a), B(a)**. Besides, the differential metabolites were clustered and shown by a heatmap diagram, **Figures 4A(b), B(b)**. Results showed that the differential metabolites can be mainly divided into the following five categories, including free fatty acids (FFAs), lysophosphatidylcholine (LPC), amino acids (AAs), purine metabolites, and bile acids. FFA, a non-esterified fatty acid (FA), is not only an important energy substance for the human body, but also affects metabolism, cell growth and differentiation (28). This study showed that the levels of FFA 14:0, FFA 16:0, FFA 18:1, FFA 18:2, FFA 18:3, FFA 20:0, FFA 20:4, and FFA 22:6 were significantly higher in the PCOS-MS group than in the PCOS group, indicating that these kinds of FFAs may be involved in the onset of MS in PCOS patients. Considering that previous studies have proved that FFA is closely related to IR (29), we believed that the increase of FFA was associated with the significant increase of IR in PCOS-MS patients. Melanie Cree-Green et al. confirmed that obese girls with PCOS had similar metabolic characteristics with diabetes, showing a close correlation between IR and FFA (30), which also verified our hypothesis. In addition, oxidative enhancement of FFA inhibits glucose from entering tissue cells by altering the REDOX potential of cells and inhibiting some key enzymes responsible for glycolysis and citric acid cycle. Thus, long-term increase of FFAs can exert cytotoxic and pro-apoptotic effects on human islet cells, inhibit the activity of phosphoinositi-3 kinase, affect the insulin receptor after signal transmission system, leading to dysfunction of glucose transporter and IR (31). Therefore, high levels of FFA and IR interact together to promote the occurrence and development of MS. Studies have found that the level of FFA 16:0 (palmitoleic acid) in plasma and follicular fluid of obese PCOS patients was higher than that of the control group and non-obese PCOS patients. For instance, Ni et al. demonstrated that FFA18:3 can predict the future development of MS in obese people (32), which were consistent with our findings. Therefore, clinical screening of the above FFAs helps us to predict the risk of MS in PCOS patients in the early stage.

Carnitine is a vital biofactor in FA oxidation responsible for the transportation of long-chain FAs from cytoplasm to the mitochondria. Acylcarnitine is transported to the mitochondria by acylbase and carnitine esters derived from the long chain FA, where acylcarnitines are changed into acylCoA and act as the substrate for *β*-oxidation. Moreover, it eliminates intracellular acyl-compounds, regulating the ratio of coenzyme A (CoA) to acyl-CoA (33). Thus serum carnitine may be an important indicator of metabolic dysfunction. A recent study conducted by Mihalik et al. (34) showed that the increase and accumulation of acylcoa intermediates in T2DM and diet-induced obesity exceeded the ability of the mitochondria to complete FA *β*-oxidation, resulting in high carnitine ester flow, and was significantly correlated with long-chain acylcarnitine levels. Our study showed that patients with PCOS-MS presented mainly with an increase in long-chain carnitine, suggesting a decrease in the oxidation of FAs, which was in line with the increase in a variety of FFAs in our study. Besides, long-chain carnitine is also associated with abnormal glucose metabolism. Melanie Cree-Gree et al. found that C16:1 and C18:1 were strongly associated with IR in obese girls with PCOS (30). Long chain acylcarnitine induces the secretion of proinflammatory factors in a concentration-dependent manner in mice. Meanwhile, long chain acylcarnitine can activate toll-like receptor on the cell surface and nucleotide binding oligomerization domain proteins, which can activate inflammation response and thus impair insulin signal transduction, leading to systemic IR (35). Besides, the short chain carnitines were also increased, such as C4:0 and C5:0, which may be due to sample collection under fasting conditions as they tend to go up in response to starvation (36).

In addition, this study showed that the serum LPC level was significantly higher in the PCOS-MS group than that in the PCOS group. LPC is not only the vital components of cell structure but also a very important medium for cellular communication, which can activate specific membrane receptors and/or nuclear receptors, involved in the development of various diseases, such as diabetes, obesity, atherosclerosis and cancer (37). LPC can induce inflammation, increase oxidative stress, and interfere with vascular endothelial function. Meanwhile, studies have displayed that LPC 16:0, 18:0, and 18:1 can induce mitochondria ROS (mtROS) in human aortic endothelial cell. In addition, studies found that mtROS is involved in LPC-induced endothelial cell activation (38). This oxidative stress and endothelial activation are also associated with PCOS and IR. Victor et al. conducted a prospective study recruiting 101 PCOS and 105 control subjects. The results showed that ROS and myeloperoxidase levels were generally increased in PCOS, especially in patients with IR (39). Moreover, they further confirmed that LPC, as a signal activator, mediated NLRP3 inflammatory activation of adipose cells induced by homocysteine, and mediated IR (40). In our study, patients with PCOS-MS had high level of IR, which may also be related to high level of LPC. Dagmar Drogan et al. (41) also found that LPC was clearly related to T2DM. Therefore, LPC possesses a good predictive and diagnostic value for PCOS combined with MS, and the pathological mechanism of LPC’s involvement in the development of MS in patients with PCOS remains to be further explored.

Furthermore, we also found that the level of branched chain amino acids (BCAAs) and aromatic amino acids (AAAs) were significantly increased in PCOS-MS group. This was consistent with the metabolomic study in follicular fluid by Zhang et al. They found that the levels of BCAAs, glutamate, and phenylalanine were increased with BMI. Moreover, leucine, valine, and glutamate were higher in PCOS with IR group than in non-IR PCOS patients and HCs (42). Tang, et al. (43) proved that decreases of BCAAs, AAAs (phenylalanine and tyrosine), and lysine in PCOS were associated with improvement in weight and insulin sensitivity. Thus, BCAAs and AAAs tend to be closely related to the occurrence of metabolic diseases. A study involving 1,302 people showed higher levels of BCAA, which was associated with MS, as well as higher levels of obesity, dyslipidemia, hypertension, and uric acid (44). Therefore, focusing on the amino acid metabolism of PCOS patients, especially the metabolism of BCAAs and AAAs, can enable us to detect the risk of MS in PCOS patients in advance and prevent various complications.

#### Differential Metabolites Between PCOS-MS Group and HC Group

Eighty-six differential metabolites were identified in the PCOS + MS group when compared with the HC group as shown in **Table 3**. The related volcano plot and heatmap diagram were respectively shown in **Figures 4A, B**. Carnitine, phospholipids, and sphingomyelins were significantly increased in PCOS-MS when compared with the HC group, but there was no statistical difference between HC and PCOS or between PCOS and PCOS-MS. It can be seen that these types of substances show a cumulative effect in the development of the disease, from health to PCOS-MS, which is worthy of note. Phospholipid is the main component of a biofilm. In eukaryotic cells, PC is produced from phosphatidylethanolamine (PE) (45), in which LPC is an intermediate product of PC. LPC can react with acylCoA transferase in liver microparticles to produce PC (46). Besides, PC/LPC ratio in serum reflects inflammatory or infectious diseases of the liver. Studies have found that LPC levels are lower in patients with drug-induced liver injury, viral hepatitis, and non-alcoholic steatohepatitis (47). Therefore, the observation of characteristics of phospholipid spectrum in patients with PCOS-MS may reflect the inflammatory state of the liver, which is consistent with the study of Li et al. (48).

Sphingolipids are also part of membrane lipids (2–20%) and are involved in a variety of eukaryotic biological processes, such as cell proliferation, differentiation, apoptosis, and so on (49). Sphingolipids can be hydrolyzed into ceramide under the action of sphingomyelinase, and ceramide plays an important role in IR (50). A large amount of ceramide can reduce insulin sensitivity and induce apoptosis of islet cells in T2DM rats (51). In addition, ceramide can promote the release of inflammatory substances such as arachidonic acid, prostaglandin, and leukotriene by activating phospholipase A2, induce the expression of various inflammation-related proteins and promote the production of Reactive Oxygen Species (ROS) at the same time (52). Therefore, high levels of sphingomyelin in PCOS patients may be associated with the pathomechanism of IR, chronic inflammation, and oxidative stress, leading to a high risk of obesity, fatty liver, and CVD (53). Therefore, the changes of SM 34:1, SM 36:2 and SM (D18:1/14:0) in PCOS patients are of positive significance for preventing the occurrence of the above complications.

### KEGG Annotation of Differential Metabolites and Pathway Analysis

**Figure 5:**
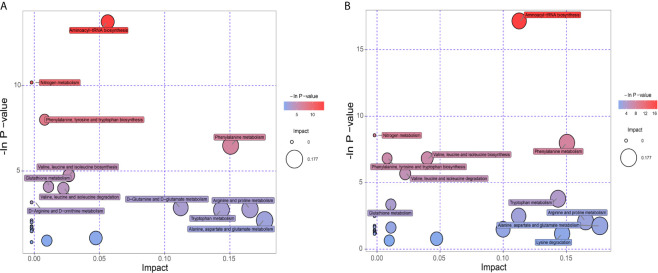
Pathway analysis for group PCOS *vs*. PCOS-MS **(A)** and group HC *vs.* PCOS-MS **(B)**.

KEGG database was utilized to annotate pathways for differential metabolites in the PCOS-MS group when compared with the PCOS or HC group. The results were shown in **Supplementary Table 1**. These differential metabolites were involved in a variety of pathways, including energy metabolism, substance transport, signaling, cell cycle regulation, and so on. In addition, we further found the core pathways with the highest correlation through comprehensive analysis including enrichment analysis and topological analysis. The results were shown in **Supplementary Table 2**. There were respectively 23 and 27 core pathways in PCOS-MS *vs*, PCOS and PCOS-MS *vs*. HC, respectively. The first 12 pathways are presented in the bubble diagram as shown in **Figures 5A, B**.

According to our findings, some pathways were both enriched in PCOS-MS *vs*. HC and PCOS-MS *vs*. PCOS, but not in PCOS *vs*. HC, such as aminoyl-tRNA biosynthesis, nitrogen metabolism, phenylalanine, tyrosine and tryptophan biosynthesis, *etc.* We projected that these pathways contributed to the onset of MS. The most significant signaling pathway was aminoacyl-tRNA biosynthesis. Aminoacyl-tRNA derives from the combination of the carboxyl of AAs and the hydroxyl of the homologous tRNA under the action of aminoacyl-tRNA synthase, which plays an important role in the synthesis of protein transport from AAs to ribosomes (54). Kyle Mohler et al. found that the aminoacylation state of the tRNA pool regulated the targets of the general amino acid control (GAAC) and targets of rapamycin (TOR) response pathway in the yeast, playing a key role in determining the accuracy and sensitivity of the stress response (55). In addition, with the development of genomics, proteomics, and functionology, other biological functions of aminoacyl-tRNA biosynthesis have been gradually revealed and used as targets for drug intervention in the treatment of various diseases, including cancer, neuropathology, autoimmune diseases, and metabolic disorders (56). For example, Lm T H et al. found that mitochondrial leucine tRNA synthase (Mito-LRS) was accumulated in T2DM and was closely related to the onset of the disease (57). The aminoacyl-tRNA synthase interaction factor (AIMP1) exerted a significant effect on regulating glucose homeostasis (58). These pieces of evidence have proved the regulatory role of aminoacyl biosynthesis in glucose metabolism. In our study, we found that the aminoacyl tRNA biosynthetic pathway was abnormal in PCOS-MS patients whether compared with the PCOS or HC groups, which may be the reason for the further development of MS in PCOS patients. In addition, considering that aminoacyl tRNA biosynthesis has been found to be closely related to the occurrence of cancer, it may also increase the risk of tumor disease in PCOS-MS patients. Thus, our findings provide implications for the future research. Clarifying the mechanism of aminoacyl- tRNA biosynthesis during the development of PCOS into MS tends to help identify accurate markers for disease screening and targeted therapies.

In addition, we also found that the PCOS-MS group showed significant disorder in AA metabolism when compared with the PCOS group, and six of the 12 pathways were related to AA metabolism, suggesting that AA metabolism plays a core part in the occurrence and development of MS in PCOS. Cross-sectional analyses of large prospective cohort studies have shown that BCAAs and AAAs are positively correlated with BMI, WC, visceral fat, SBP, DBP, FBG, FINS, and TG, while negatively correlated with HDL-C (59). Moreover, accumulated evidence revealed that the abundant bacteria responsible for AA fermentation in the large intestine have a huge impact on host metabolism, immunity, reproduction, and other functions. Zeng et al. confirmed that in MS mice, the relative abundance of Firmicutes increased significantly, and Firmicutes-to-Bacteroidetes ratio was higher, which was consistent with the occurrence of obesity, hyperlipidemia, IR, and other phenotypes (60). This also suggests multi-omics analysis can be taken into consideration in future to further explore the association between AA metabolism and MS in PCOS from multiple perspectives. Due to significantly increased levels of various types of AAs and their abnormal metabolism in PCOS-MS, we can pay special attention to guiding patients to change their diet structure and reduce the intake of certain AAs. Meanwhile, probiotics can be appropriately supplemented to improve the composition of gut microbiota and amino acid metabolism. For example, bacteroides by gavage can reduce serum BCAA concentration in mice and improve diet-induced obesity (61). Thus, pathway analysis provides us new direction for treatment.

### Network Analysis for Differential Metabolites of Group PCOS + MS *vs.* PCOS

**Figure 6:**
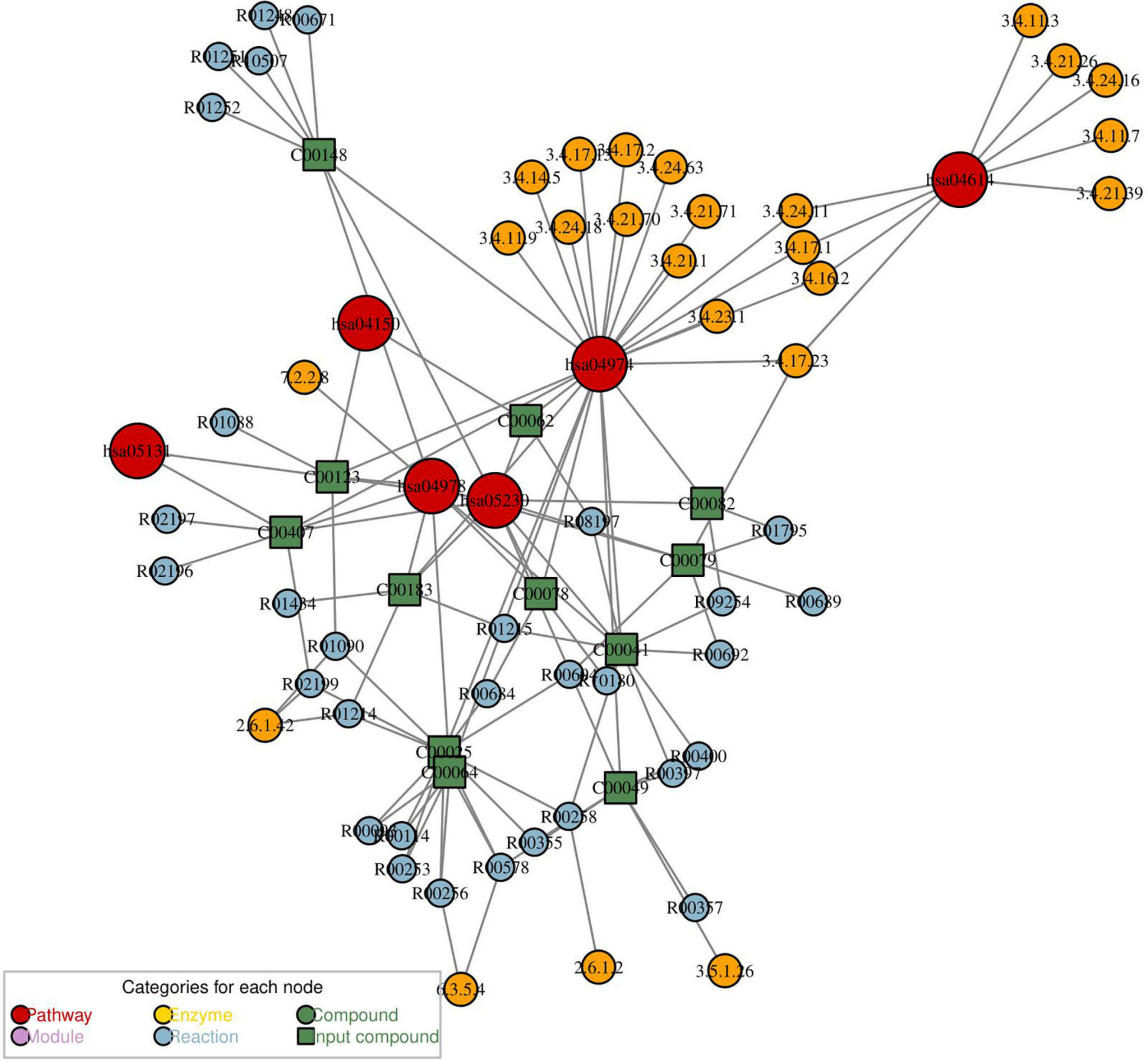
The metabolic network analysis for differential metabolites of PCOS *vs*. PCOS-MS. In this diagram, the red dot represented a metabolic pathway; yellow dot represents enzyme; green dot represents background material; blue dot represents a chemical reaction; and a green square represents the difference of materials.

Network analysis for differential metabolites of group PCOS + MS *vs*. PCOS was constructed with “FELLA” package in R language. This network can reflect the interaction of pathways, enzymes, and metabolites as shown in **Figure 6**. We obtained five pathways, including the mammalian target of rapamycin (mTOR) signaling pathway, renin–angiotensin system, protein digestion and absorption, mineral absorption, central carbon metabolism in cancer, and Shigellosis. They interacted with a variety of enzymes and regulated various biological processes in PCOS-MS. The mTOR pathway, for example, can affect cell growth, proliferation, and survival by activating ribosomal kinase (62). The figure showed its interaction with BCAA transaminase and participation in L-Leucine: 2-oxoglutarate aminotransferase reaction. BCAAs belong to essential AAs and can only be obtained from dietary sources. Studies have proved that high level of BCAAs in plasma can continuously activate the mTOR signaling pathway and disassociate insulin receptor from insulin receptor substrate 1, which is closely related to the occurrence of T2DM and obesity (63). A study proved that ketoisocaproic acid, a metabolite of leucine, suppressed insulin-stimulated glucose transport in skeletal muscle cells (64). Conversely, deprivation of any BCAAs in normal weekly diet or restriction of all the three BCAAs in genetically diabetic Zucker rats with isocaloric and isonitrogen diets could significantly improve insulin sensitivity and glycemic control (65). We hypothesized that the occurrence of IR in PCOS-MS may be related to the increase of BCAAs and the overactivation of mTOR. Song et al. demonstrated that mTORC1 can be activated by DHEA in the PCOS mice, leading to IR (66). These studies were consistent with our predictions. In addition, we also found that caspase-4 and caspase-11, which are related to apoptosis, are also involved in the inhibition of autophagy mediated by mTOR. Various studies have reported that autophagy and apoptosis are two forms of cell death, and their relationship is complex and subtle. On one hand, autophagy can inhibit apoptosis under certain environmental stress. However, excessive depletion of intracellular substance caused by autophagy can lead to cell apoptosis (67). mTOR is a pivotal molecule that regulates autophagy, and the overactivation of mTOR in PCOS-MS patients may affect the balance between autophagy and apoptosis, thus leading to complex pathological mechanism in patients with PCOS-MS. Last but not least, the figure also showed other metabolic pathways and regulatory mechanisms, which were complementary and mutually corroborated with the results of our previous pathway analysis. Furthermore, this network is helpful to provide targets for drug intervention, leading to advances in treatment.

### Clinical Implications From Metabonomics Analysis

#### Correlation Analysis of Differential Metabolites and Clinical Indicators in PCOS-MS *vs.* PCOS Groups

**Figure 7:**
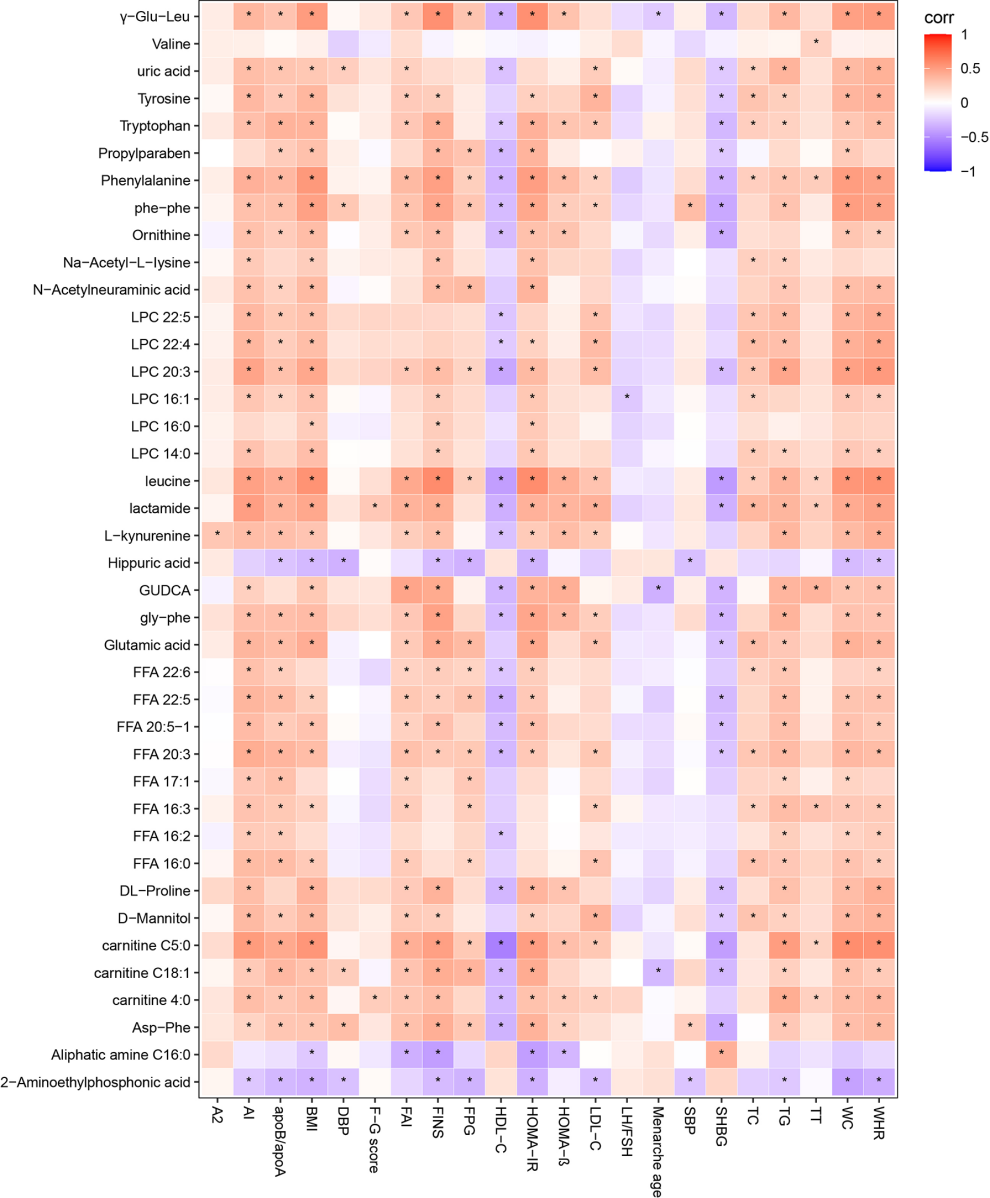
Correlation heatmap of differential metabolites and differential clinical indicators in PCOS *vs*. PCOS-MS. In this picture, red represents corr = 1, blue represents corr = –1, and white represents corr = 0. “*” in the figure represents *P* < 0.05. The X-axis stands for differential metabolomics, and the Y-axis stands for differential clinical data in PCOS *vs*. PCOS-MS.

Spearman correlation was calculated for the differential metabolites and clinical indicators between PCOS-MS and PCOS groups. The correlation coefficient (Corr) matrix and the correlation *P* value were obtained, and the matrix was used for subsequent analysis and production of heatmap. In the correlation analysis, we found 397 pairs of significantly correlated differential metabolites and clinical indications (*P <* 0.05), and the correlation coefficient of 17 pairs was greater than 0.4, involving 11 metabolites that tend to be biomarkers in clinical practice as shown in **Supplementary Table 3** and **Figure 7**. Carnitine in patients with PCOS-MS is closely related to glycolipid metabolism and reproductive hormones. For example, carnitine C18:1 (0.4310) is closely related to glucose metabolism indicators such as HOMA-IR (0.4310), FINS (0.4221), and carnitine C4:0 is closely associated with TG (0.426). The results are consistent with our previous analysis. Besides, the close correlation of carnitine and reproductive hormone metabolism is also discovered. For example, carnitine C5:0 is negatively correlated with SHBG and positively correlated with FAI. This is consistent with the Vigerust and others’ research, and they also found that total and free carnitines were negatively correlated with SHBG in PCOS patients (68).

#### Validation of the Combinational Metabolic Biomarkers

**Figure 8:**
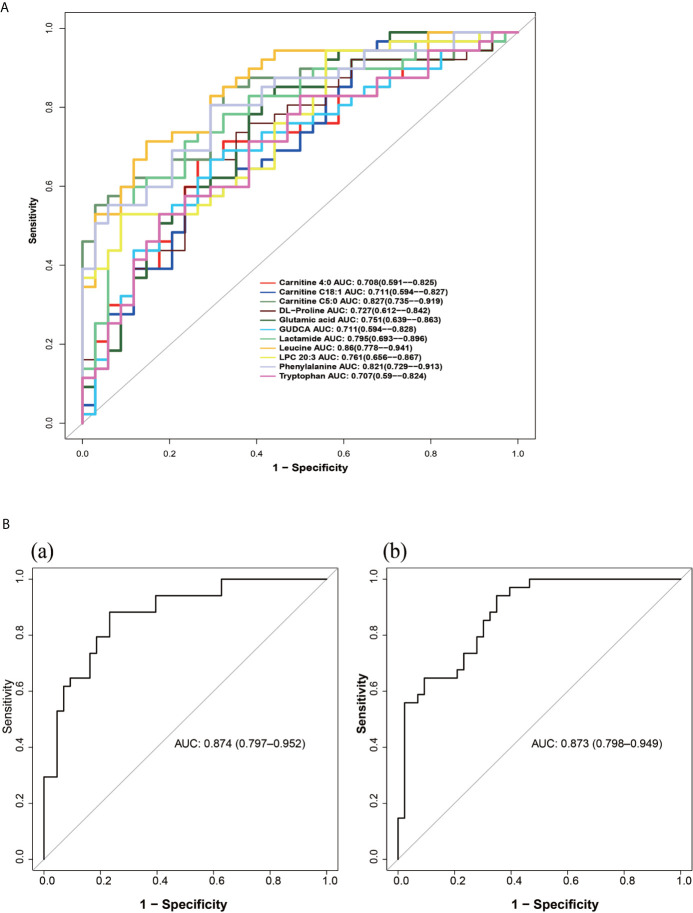
**(A)** The ROC curve of single metabolites as potential biomarkers for the diagnosis of MS in patients with PCOS. **(B)** The ROC curves of metabolite composition as potential biomarkers, (a) glutamic acid + leucine + phenylalanine; (b) carnitine C 4:0 + carnitine C18:1 + carnitine C5:0.

We evaluated the diagnostic value of 11 metabolites closely related to the clinical manifestations of PCOS-MS by “pPOC” package in R language as shown in **Figures 8A, B**. Among them, biomarkers with high diagnostic value were leucine, carnitine C5:0, phenylalanine, lactamide, LPC 20:3, *etc* (**Figure 8A**). To further improve the diagnosis sensitivity, we combined multiple biomarkers as a sensitive screening index for PCOS-MS. Glutamic acid + leucine + phenylalanine and carnitine C 4:0 + carnitine C18:1 + carnitine C5:0 were expected to be a sensitive indicator of disease screening, with AUC of 0.874 and 0.873, respectively as shown in **Figures 8B(a), B(b)**. The cut-off values were respectively 0.375 (specificity = 0.767, sensitivity = 0.882) and 0.282 (specificity = 0.651, sensitivity = 0.941).

## Conclusion

PCOS is a long-term challenge in clinical and basic research studies which aim to clarify the origin, pathological mechanism, and development trend of PCOS. In this study, we compared the clinical characteristics of HC, PCOS, and PCOS-MS patients, and then analyzed the metabolic characteristics of PCOS-MS when compared with HC and PCOS respectively. KEGG annotation, pathway analysis, and metabolic network analysis of the differential metabolites between PCOS and PCOS-MS helped us to identify the metabolic mechanism of PCOS development into MS. In addition, we also analyzed the correlation between differential metabolites and clinical indicators and evaluated their diagnostic value with ROC curve so as to find sensitive combinational biomarkers in clinical practice. On considering that techniques utilized in metabolomic studies have matured, attempts have been made to look beyond the potential for biomarker identification and begin to understand the role metabolites might have in disease pathogenesis. So in our next research project, we expect to combine metabolomics with genomics to further explore the significance of differential metabolites in pathogenesis of PCOS-MS. This method—triangulation of genetic, metabolomic, and phenotypic data applied to the MS state in PCOS—is expected to be a potent technique for determining the contribution of metabolite biomarkers to disease. Besides, further effort should be made to replicate and validate these biomarkers in additional population cohorts so as to serve in the clinical work of screening at-risk population, assessing drug efficacy, lifestyle interventions, as well as potentially discovering novel therapeutic targets.

## Precis

Our study provides a new insight to understand the pathogenic mechanism and to discriminate metabolites that may help screen patients with PCOS at high-risk for MS and provide sensitive biomarkers for clinical diagnosis.

## Data Availability Statement

The original contributions presented in the study are included in the article/**Supplementary Material**. Further inquiries can be directed to the corresponding authors.

## Code Availability

SIEVE 1.2 version Workstation (Thermo Fisher Scientific, Waltham, MA, USA); SIMCA-P 11.0 version (Umetrics, Umea, Sweden); IBM SPSS 25.0 (SPSS, Chicago, IL, USA); SAS 9.3(SAS Institute Inc, Cary, NC, USA).

## Ethics Statement

The studies involving human participants were reviewed and approved by the Ethics Committee of Heilongjiang University of Traditional Chinese Medicine (NO. HZYLLKT201500401). The patients/participants provided their written informed consent to participate in this study.

## Author Contributions

X-XZ and XF data analysis, figure preparation and manuscript preparation. X-JZ, YJ and XL interpretation, data analyses, and manuscript submission. JN and XM data interpretation, figure preparation. JW and GX critically reviewed the manuscript. LH and YW critically reviewed the manuscript and study initiation. All authors approved the final version of the manuscript. And all the authors contributed to the article and approved the submitted version.

## Funding

This work was supported by the National Natural Science Foundation of China (grant number 81904235;81973894), the Project of General Undergraduate University Youth Innovation Talents by Education Department of Heilongjiang Province (grant number UNPYSCT-2018227), and the Project of Excellent Innovation Talents by Heilongjiang University of Chinese Medicine (grant number 2018RCQ03).

## Conflict of Interest

The authors declare that the research was conducted in the absence of any commercial or financial relationships that could be construed as a potential conflict of interest.

## Abbreviation

PCOS, polycystic ovary syndrome; MS, metabolic syndrome; UPLC, ultraperformance liquid chromatography; ROC, the receiver operating characteristic; T2DM, type 2 diabetes; CVD, cardiovascular disease; BMI, body mass index; FINS, fasting insulin; HDL, high density lipoprotein; HC, healthy control; WC, waist circumference; TG, triglyceride; SBP, systolic blood pressure; DBP, diastolic blood pressure; FBG, fasting blood glucose; FSH, follicle stimulating hormone; LH, luteinizing hormone; TT, total testosterone; DHEAS, dehydroepiandrosterone sulfate; A2, androstenedione; SHBG, sex hormone binding globulin; HOMA-IR, homeostatic model index of insulin resistance; TC, total cholesterol; LDL-C, low density lipoprotein cholesterol; FAI, free androgen index; AI, atherosclerotic index; WHR, waist to hip ratio; F–G score, Ferriman–Gallwey score; ESI, electrospray ionization; PCA, principal component analysis; OPLS-DA, orthogonal partial least squares discriminant analysis; VIP, Variable importance in the projection; KEGG, Kyoto Encyclopedia of Genes and Genomes; FFA, free fatty acid; AAs, amino acids; LPC, lysophosphatidylcholine; BCAA, branched chain amino acid; AAA, aromatic amino acid; FA, fatty acid.
