# Supplementary figures and images for: How to Screen and Prevent Metabolic Syndrome in Patients of PCOS Early: Implications From Metabolomics
Source: Front Endocrinol (Lausanne). 2021 Jun 2;12:659268. doi: 10.3389/fendo.2021.659268 (PMC8207510; doi:10.3389/fendo.2021.659268)

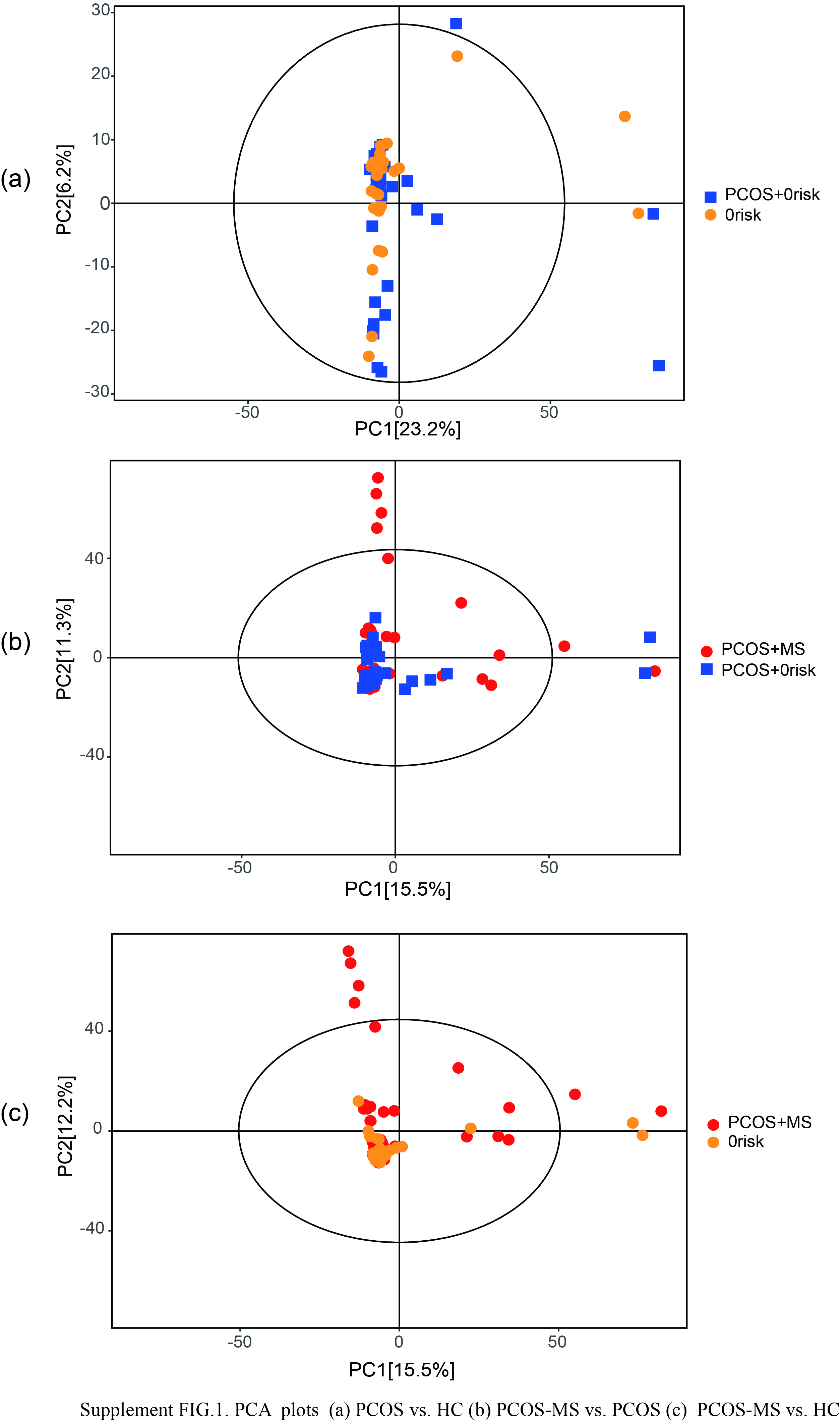

Supplement: Supplementary file 1 [file Image_1.jpeg]
