# Supplementary material for: How to Screen and Prevent Metabolic Syndrome in Patients of PCOS Early: Implications From Metabolomics
Source: Front Endocrinol (Lausanne). 2021 Jun 2;12:659268. doi: 10.3389/fendo.2021.659268 (PMC8207510; doi:10.3389/fendo.2021.659268)
Supplement: Supplementary file 2 [file Table_1.docx]

**Supplement Table 1. Annotation information of KEGG pathways for differential metabolites between groups**

1. **PCOS vs. PCOS-MS**

| **KEGG pathway** | **Compound** |
| --- | --- |
| hsa01100 Metabolic pathways - Homo sapiens (human)(12) | cpd:C03557 2-Aminoethylphosphonate; cpd:C00392 Mannitol; cpd:C00025 L-Glutamate; cpd:C01586 Hippurate; cpd:C00123 L-Leucine; cpd:C00328 L-Kynurenine; cpd:C00077 L-Ornithine; cpd:C00079 L-Phenylalanine; cpd:C00078 L-Tryptophan; cpd:C00082 L-Tyrosine; cpd:C00366 Urate; cpd:C00183 L-Valine |
| hsa01210 2-Oxocarboxylic acid metabolism - Homo sapiens (human)(7) | cpd:C00025 L-Glutamate; cpd:C00123 L-Leucine; cpd:C00077 L-Ornithine; cpd:C00079 L-Phenylalanine; cpd:C00078 L-Tryptophan; cpd:C00082 L-Tyrosine; cpd:C00183 L-Valine |
| hsa01230 Biosynthesis of amino acids - Homo sapiens (human)(7) | cpd:C00025 L-Glutamate; cpd:C00123 L-Leucine; cpd:C00077 L-Ornithine; cpd:C00079 L-Phenylalanine; cpd:C00078 L-Tryptophan; cpd:C00082 L-Tyrosine; cpd:C00183 L-Valine |
| hsa02010 ABC transporters - Homo sapiens (human)(7) | cpd:C03557 2-Aminoethylphosphonate; cpd:C00392 Mannitol; cpd:C00025 L-Glutamate; cpd:C00123 L-Leucine; cpd:C00077 L-Ornithine; cpd:C00079 L-Phenylalanine; cpd:C00183 L-Valine |
| hsa00970 Aminoacyl-tRNA biosynthesis - Homo sapiens (human)(6) | cpd:C00025 L-Glutamate; cpd:C00123 L-Leucine; cpd:C00079 L-Phenylalanine; cpd:C00078 L-Tryptophan; cpd:C00082 L-Tyrosine; cpd:C00183 L-Valine |
| hsa04974 Protein digestion and absorption - Homo sapiens (human)(6) | cpd:C00025 L-Glutamate; cpd:C00123 L-Leucine; cpd:C00079 L-Phenylalanine; cpd:C00078 L-Tryptophan; cpd:C00082 L-Tyrosine; cpd:C00183 L-Valine |
| hsa05230 Central carbon metabolism in cancer - Homo sapiens (human)(6) | cpd:C00025 L-Glutamate; cpd:C00123 L-Leucine; cpd:C00079 L-Phenylalanine; cpd:C00078 L-Tryptophan; cpd:C00082 L-Tyrosine; cpd:C00183 L-Valine |
| hsa04978 Mineral absorption - Homo sapiens (human)(4) | cpd:C00123 L-Leucine; cpd:C00079 L-Phenylalanine; cpd:C00078 L-Tryptophan; cpd:C00183 L-Valine |
| hsa00360 Phenylalanine metabolism - Homo sapiens (human)(3) | cpd:C01586 Hippurate; cpd:C00079 L-Phenylalanine; cpd:C00082 L-Tyrosine |
| hsa00400 Phenylalanine, tyrosine and tryptophan biosynthesis - Homo sapiens (human)(3) | cpd:C00079 L-Phenylalanine; cpd:C00078 L-Tryptophan; cpd:C00082 L-Tyrosine |
| hsa00220 Arginine biosynthesis - Homo sapiens (human)(2) | cpd:C00025 L-Glutamate; cpd:C00077 L-Ornithine |
| hsa00280 Valine, leucine and isoleucine degradation - Homo sapiens (human)(2) | cpd:C00123 L-Leucine; cpd:C00183 L-Valine |
| hsa00290 Valine, leucine and isoleucine biosynthesis - Homo sapiens (human)(2) | cpd:C00123 L-Leucine; cpd:C00183 L-Valine |
| hsa00330 Arginine and proline metabolism - Homo sapiens (human)(2) | cpd:C00025 L-Glutamate; cpd:C00077 L-Ornithine |
| hsa00380 Tryptophan metabolism - Homo sapiens (human)(2) | cpd:C00328 L-Kynurenine; cpd:C00078 L-Tryptophan |
| hsa00480 Glutathione metabolism - Homo sapiens (human)(2) | cpd:C00025 L-Glutamate; cpd:C00077 L-Ornithine |
| hsa05030 Cocaine addiction - Homo sapiens (human)(2) | cpd:C00025 L-Glutamate; cpd:C00082 L-Tyrosine |
| hsa05031 Amphetamine addiction - Homo sapiens (human)(2) | cpd:C00025 L-Glutamate; cpd:C00082 L-Tyrosine |
| hsa05034 Alcoholism - Homo sapiens (human)(2) | cpd:C00025 L-Glutamate; cpd:C00082 L-Tyrosine |
| hsa05143 African trypanosomiasis - Homo sapiens (human)(2) | cpd:C00328 L-Kynurenine; cpd:C00078 L-Tryptophan |
| hsa00051 Fructose and mannose metabolism - Homo sapiens (human)(1) | cpd:C00392 Mannitol |
| hsa00130 Ubiquinone and other terpenoid-quinone biosynthesis - Homo sapiens (human)(1) | cpd:C00082 L-Tyrosine |
| hsa00230 Purine metabolism - Homo sapiens (human)(1) | cpd:C00366 Urate |
| hsa00250 Alanine, aspartate and glutamate metabolism - Homo sapiens (human)(1) | cpd:C00025 L-Glutamate |
| hsa00260 Glycine, serine and threonine metabolism - Homo sapiens (human)(1) | cpd:C00078 L-Tryptophan |
| hsa00340 Histidine metabolism - Homo sapiens (human)(1) | cpd:C00025 L-Glutamate |
| hsa00350 Tyrosine metabolism - Homo sapiens (human)(1) | cpd:C00082 L-Tyrosine |
| hsa00430 Taurine and hypotaurine metabolism - Homo sapiens (human)(1) | cpd:C00025 L-Glutamate |
| hsa00440 Phosphonate and phosphinate metabolism - Homo sapiens (human)(1) | cpd:C03557 2-Aminoethylphosphonate |
| hsa00471 D-Glutamine and D-glutamate metabolism - Homo sapiens (human)(1) | cpd:C00025 L-Glutamate |
| hsa00472 D-Arginine and D-ornithine metabolism - Homo sapiens (human)(1) | cpd:C00077 L-Ornithine |
| hsa00524 Neomycin, kanamycin and gentamicin biosynthesis - Homo sapiens (human)(1) | cpd:C00025 L-Glutamate |
| hsa00630 Glyoxylate and dicarboxylate metabolism - Homo sapiens (human)(1) | cpd:C00025 L-Glutamate |
| hsa00650 Butanoate metabolism - Homo sapiens (human)(1) | cpd:C00025 L-Glutamate |
| hsa00730 Thiamine metabolism - Homo sapiens (human)(1) | cpd:C00082 L-Tyrosine |
| hsa00770 Pantothenate and CoA biosynthesis - Homo sapiens (human)(1) | cpd:C00183 L-Valine |
| hsa00860 Porphyrin and chlorophyll metabolism - Homo sapiens (human)(1) | cpd:C00025 L-Glutamate |
| hsa00910 Nitrogen metabolism - Homo sapiens (human)(1) | cpd:C00025 L-Glutamate |
| hsa01200 Carbon metabolism - Homo sapiens (human)(1) | cpd:C00025 L-Glutamate |
| hsa04068 FoxO signaling pathway - Homo sapiens (human)(1) | cpd:C00025 L-Glutamate |
| hsa04072 Phospholipase D signaling pathway - Homo sapiens (human)(1) | cpd:C00025 L-Glutamate |
| hsa04080 Neuroactive ligand-receptor interaction - Homo sapiens (human)(1) | cpd:C00025 L-Glutamate |
| hsa04150 mTOR signaling pathway - Homo sapiens (human)(1) | cpd:C00123 L-Leucine |
| hsa04216 Ferroptosis - Homo sapiens (human)(1) | cpd:C00025 L-Glutamate |
| hsa04540 Gap junction - Homo sapiens (human)(1) | cpd:C00025 L-Glutamate |
| hsa04713 Circadian entrainment - Homo sapiens (human)(1) | cpd:C00025 L-Glutamate |
| hsa04720 Long-term potentiation - Homo sapiens (human)(1) | cpd:C00025 L-Glutamate |
| hsa04721 Synaptic vesicle cycle - Homo sapiens (human)(1) | cpd:C00025 L-Glutamate |
| hsa04723 Retrograde endocannabinoid signaling - Homo sapiens (human)(1) | cpd:C00025 L-Glutamate |
| hsa04724 Glutamatergic synapse - Homo sapiens (human)(1) | cpd:C00025 L-Glutamate |
| hsa04726 Serotonergic synapse - Homo sapiens (human)(1) | cpd:C00078 L-Tryptophan |
| hsa04727 GABAergic synapse - Homo sapiens (human)(1) | cpd:C00025 L-Glutamate |
| hsa04728 Dopaminergic synapse - Homo sapiens (human)(1) | cpd:C00082 L-Tyrosine |
| hsa04730 Long-term depression - Homo sapiens (human)(1) | cpd:C00025 L-Glutamate |
| hsa04742 Taste transduction - Homo sapiens (human)(1) | cpd:C00025 L-Glutamate |
| hsa04916 Melanogenesis - Homo sapiens (human)(1) | cpd:C00082 L-Tyrosine |
| hsa04917 Prolactin signaling pathway - Homo sapiens (human)(1) | cpd:C00082 L-Tyrosine |
| hsa04964 Proximal tubule bicarbonate reclamation - Homo sapiens (human)(1) | cpd:C00025 L-Glutamate |
| hsa04976 Bile secretion - Homo sapiens (human)(1) | cpd:C00366 Urate |
| hsa05012 Parkinson disease - Homo sapiens (human)(1) | cpd:C00082 L-Tyrosine |
| hsa05014 Amyotrophic lateral sclerosis (ALS) - Homo sapiens (human)(1) | cpd:C00025 L-Glutamate |
| hsa05016 Huntington disease - Homo sapiens (human)(1) | cpd:C00025 L-Glutamate |
| hsa05033 Nicotine addiction - Homo sapiens (human)(1) | cpd:C00025 L-Glutamate |

1. HC vs. PCOS-MS

| **KEGG pathway** | **Compound** |
| --- | --- |
| hsa01100 Metabolic pathways - Homo sapiens (human)(14) | cpd:C00486 Bilirubin; cpd:C00392 Mannitol; cpd:C00025 L-Glutamate; cpd:C01586 Hippurate; cpd:C00123 L-Leucine; cpd:C00407 L-Isoleucine; cpd:C00328 L-Kynurenine; cpd:C00047 L-Lysine; cpd:C00077 L-Ornithine; cpd:C00079 L-Phenylalanine; cpd:C00078 L-Tryptophan; cpd:C00082 L-Tyrosine; cpd:C00366 Urate; cpd:C00183 L-Valine |
| hsa01210 2-Oxocarboxylic acid metabolism - Homo sapiens (human)(9) | cpd:C00025 L-Glutamate; cpd:C00123 L-Leucine; cpd:C00407 L-Isoleucine; cpd:C00047 L-Lysine; cpd:C00077 L-Ornithine; cpd:C00079 L-Phenylalanine; cpd:C00078 L-Tryptophan; cpd:C00082 L-Tyrosine; cpd:C00183 L-Valine |
| hsa01230 Biosynthesis of amino acids - Homo sapiens (human)(9) | cpd:C00025 L-Glutamate; cpd:C00123 L-Leucine; cpd:C00407 L-Isoleucine; cpd:C00047 L-Lysine; cpd:C00077 L-Ornithine; cpd:C00079 L-Phenylalanine; cpd:C00078 L-Tryptophan; cpd:C00082 L-Tyrosine; cpd:C00183 L-Valine |
| hsa00970 Aminoacyl-tRNA biosynthesis - Homo sapiens (human)(8) | cpd:C00025 L-Glutamate; cpd:C00123 L-Leucine; cpd:C00407 L-Isoleucine; cpd:C00047 L-Lysine; cpd:C00079 L-Phenylalanine; cpd:C00078 L-Tryptophan; cpd:C00082 L-Tyrosine; cpd:C00183 L-Valine |
| hsa02010 ABC transporters - Homo sapiens (human)(8) | cpd:C00392 Mannitol; cpd:C00025 L-Glutamate; cpd:C00123 L-Leucine; cpd:C00407 L-Isoleucine; cpd:C00047 L-Lysine; cpd:C00077 L-Ornithine; cpd:C00079 L-Phenylalanine; cpd:C00183 L-Valine |
| hsa04974 Protein digestion and absorption - Homo sapiens (human)(8) | cpd:C00025 L-Glutamate; cpd:C00123 L-Leucine; cpd:C00407 L-Isoleucine; cpd:C00047 L-Lysine; cpd:C00079 L-Phenylalanine; cpd:C00078 L-Tryptophan; cpd:C00082 L-Tyrosine; cpd:C00183 L-Valine |
| hsa05230 Central carbon metabolism in cancer - Homo sapiens (human)(7) | cpd:C00025 L-Glutamate; cpd:C00123 L-Leucine; cpd:C00407 L-Isoleucine; cpd:C00079 L-Phenylalanine; cpd:C00078 L-Tryptophan; cpd:C00082 L-Tyrosine; cpd:C00183 L-Valine |
| hsa04978 Mineral absorption - Homo sapiens (human)(5) | cpd:C00123 L-Leucine; cpd:C00407 L-Isoleucine; cpd:C00079 L-Phenylalanine; cpd:C00078 L-Tryptophan; cpd:C00183 L-Valine |
| hsa00360 Phenylalanine metabolism - Homo sapiens (human)(4) | cpd:C01586 Hippurate; cpd:C04148 Phenylacetylglutamine; cpd:C00079 L-Phenylalanine; cpd:C00082 L-Tyrosine |
| hsa00280 Valine, leucine and isoleucine degradation - Homo sapiens (human)(3) | cpd:C00123 L-Leucine; cpd:C00407 L-Isoleucine; cpd:C00183 L-Valine |
| hsa00290 Valine, leucine and isoleucine biosynthesis - Homo sapiens (human)(3) | cpd:C00123 L-Leucine; cpd:C00407 L-Isoleucine; cpd:C00183 L-Valine |
| hsa00400 Phenylalanine, tyrosine and tryptophan biosynthesis - Homo sapiens (human)(3) | cpd:C00079 L-Phenylalanine; cpd:C00078 L-Tryptophan; cpd:C00082 L-Tyrosine |
| hsa04976 Bile secretion - Homo sapiens (human)(3) | cpd:C00486 Bilirubin; cpd:C00318 L-Carnitine; cpd:C00366 Urate |
| hsa00220 Arginine biosynthesis - Homo sapiens (human)(2) | cpd:C00025 L-Glutamate; cpd:C00077 L-Ornithine |
| hsa00330 Arginine and proline metabolism - Homo sapiens (human)(2) | cpd:C00025 L-Glutamate; cpd:C00077 L-Ornithine |
| hsa00380 Tryptophan metabolism - Homo sapiens (human)(2) | cpd:C00328 L-Kynurenine; cpd:C00078 L-Tryptophan |
| hsa00480 Glutathione metabolism - Homo sapiens (human)(2) | cpd:C00025 L-Glutamate; cpd:C00077 L-Ornithine |
| hsa00860 Porphyrin and chlorophyll metabolism - Homo sapiens (human)(2) | cpd:C00486 Bilirubin; cpd:C00025 L-Glutamate |
| hsa05030 Cocaine addiction - Homo sapiens (human)(2) | cpd:C00025 L-Glutamate; cpd:C00082 L-Tyrosine |
| hsa05031 Amphetamine addiction - Homo sapiens (human)(2) | cpd:C00025 L-Glutamate; cpd:C00082 L-Tyrosine |
| hsa05034 Alcoholism - Homo sapiens (human)(2) | cpd:C00025 L-Glutamate; cpd:C00082 L-Tyrosine |
| hsa05143 African trypanosomiasis - Homo sapiens (human)(2) | cpd:C00328 L-Kynurenine; cpd:C00078 L-Tryptophan |
| hsa00051 Fructose and mannose metabolism - Homo sapiens (human)(1) | cpd:C00392 Mannitol |
| hsa00130 Ubiquinone and other terpenoid-quinone biosynthesis - Homo sapiens (human)(1) | cpd:C00082 L-Tyrosine |
| hsa00230 Purine metabolism - Homo sapiens (human)(1) | cpd:C00366 Urate |
| hsa00250 Alanine, aspartate and glutamate metabolism - Homo sapiens (human)(1) | cpd:C00025 L-Glutamate |
| hsa00260 Glycine, serine and threonine metabolism - Homo sapiens (human)(1) | cpd:C00078 L-Tryptophan |
| hsa00310 Lysine degradation - Homo sapiens (human)(1) | cpd:C00047 L-Lysine |
| hsa00340 Histidine metabolism - Homo sapiens (human)(1) | cpd:C00025 L-Glutamate |
| hsa00350 Tyrosine metabolism - Homo sapiens (human)(1) | cpd:C00082 L-Tyrosine |
| hsa00430 Taurine and hypotaurine metabolism - Homo sapiens (human)(1) | cpd:C00025 L-Glutamate |
| hsa00471 D-Glutamine and D-glutamate metabolism - Homo sapiens (human)(1) | cpd:C00025 L-Glutamate |
| hsa00472 D-Arginine and D-ornithine metabolism - Homo sapiens (human)(1) | cpd:C00077 L-Ornithine |
| hsa00524 Neomycin, kanamycin and gentamicin biosynthesis - Homo sapiens (human)(1) | cpd:C00025 L-Glutamate |
| hsa00630 Glyoxylate and dicarboxylate metabolism - Homo sapiens (human)(1) | cpd:C00025 L-Glutamate |
| hsa00650 Butanoate metabolism - Homo sapiens (human)(1) | cpd:C00025 L-Glutamate |
| hsa00730 Thiamine metabolism - Homo sapiens (human)(1) | cpd:C00082 L-Tyrosine |
| hsa00770 Pantothenate and CoA biosynthesis - Homo sapiens (human)(1) | cpd:C00183 L-Valine |
| hsa00780 Biotin metabolism - Homo sapiens (human)(1) | cpd:C00047 L-Lysine |
| hsa00910 Nitrogen metabolism - Homo sapiens (human)(1) | cpd:C00025 L-Glutamate |
| hsa01200 Carbon metabolism - Homo sapiens (human)(1) | cpd:C00025 L-Glutamate |
| hsa04068 FoxO signaling pathway - Homo sapiens (human)(1) | cpd:C00025 L-Glutamate |
| hsa04072 Phospholipase D signaling pathway - Homo sapiens (human)(1) | cpd:C00025 L-Glutamate |
| hsa04080 Neuroactive ligand-receptor interaction - Homo sapiens (human)(1) | cpd:C00025 L-Glutamate |
| hsa04150 mTOR signaling pathway - Homo sapiens (human)(1) | cpd:C00123 L-Leucine |
| hsa04216 Ferroptosis - Homo sapiens (human)(1) | cpd:C00025 L-Glutamate |
| hsa04540 Gap junction - Homo sapiens (human)(1) | cpd:C00025 L-Glutamate |
| hsa04713 Circadian entrainment - Homo sapiens (human)(1) | cpd:C00025 L-Glutamate |
| hsa04714 Thermogenesis - Homo sapiens (human)(1) | cpd:C00318 L-Carnitine |
| hsa04720 Long-term potentiation - Homo sapiens (human)(1) | cpd:C00025 L-Glutamate |
| hsa04721 Synaptic vesicle cycle - Homo sapiens (human)(1) | cpd:C00025 L-Glutamate |
| hsa04723 Retrograde endocannabinoid signaling - Homo sapiens (human)(1) | cpd:C00025 L-Glutamate |
| hsa04724 Glutamatergic synapse - Homo sapiens (human)(1) | cpd:C00025 L-Glutamate |
| hsa04726 Serotonergic synapse - Homo sapiens (human)(1) | cpd:C00078 L-Tryptophan |
| hsa04727 GABAergic synapse - Homo sapiens (human)(1) | cpd:C00025 L-Glutamate |
| hsa04728 Dopaminergic synapse - Homo sapiens (human)(1) | cpd:C00082 L-Tyrosine |
| hsa04730 Long-term depression - Homo sapiens (human)(1) | cpd:C00025 L-Glutamate |
| hsa04742 Taste transduction - Homo sapiens (human)(1) | cpd:C00025 L-Glutamate |
| hsa04916 Melanogenesis - Homo sapiens (human)(1) | cpd:C00082 L-Tyrosine |
| hsa04917 Prolactin signaling pathway - Homo sapiens (human)(1) | cpd:C00082 L-Tyrosine |
| hsa04964 Proximal tubule bicarbonate reclamation - Homo sapiens (human)(1) | cpd:C00025 L-Glutamate |
| hsa05012 Parkinson disease - Homo sapiens (human)(1) | cpd:C00082 L-Tyrosine |
| hsa05014 Amyotrophic lateral sclerosis (ALS) - Homo sapiens (human)(1) | cpd:C00025 L-Glutamate |
| hsa05016 Huntington disease - Homo sapiens (human)(1) | cpd:C00025 L-Glutamate |
| hsa05033 Nicotine addiction - Homo sapiens (human)(1) | cpd:C00025 L-Glutamate |
