# Supplementary material for: How to Screen and Prevent Metabolic Syndrome in Patients of PCOS Early: Implications From Metabolomics
Source: Front Endocrinol (Lausanne). 2021 Jun 2;12:659268. doi: 10.3389/fendo.2021.659268 (PMC8207510; doi:10.3389/fendo.2021.659268)
Supplement: Supplementary file 3 [file Table_2.docx]

**Supplement Table 2. Analysis of metabolic pathways of the different metabolites between groups**

1. **PCOS vs. PCOS-MS**

| **Pathway** | **Total** | **Hits** | **-ln(p)** | **FDR** | **Impact** |
| --- | --- | --- | --- | --- | --- |
| Aminoacyl-tRNA biosynthesis | 75 | 6 | 13.737 | 0.000086493 | 0.05634 |
| Nitrogen metabolism | 39 | 4 | 10.18 | 0.0015174 | 0 |
| Phenylalanine, tyrosine and tryptophan biosynthesis | 27 | 3 | 8.0035 | 0.0089148 | 0.008 |
| Phenylalanine metabolism | 45 | 3 | 6.4807 | 0.030655 | 0.15056 |
| Valine, leucine and isoleucine biosynthesis | 27 | 2 | 4.7375 | 0.14017 | 0.0265 |
| Glutathione metabolism | 38 | 2 | 4.0764 | 0.21385 | 0.01095 |
| Valine, leucine and isoleucine degradation | 40 | 2 | 3.9786 | 0.21385 | 0.02232 |
| D-Arginine and D-ornithine metabolism | 8 | 1 | 3.1592 | 0.4246 | 0 |
| D-Glutamine and D-glutamate metabolism | 11 | 1 | 2.8482 | 0.47748 | 0.1123 |
| Arginine and proline metabolism | 77 | 2 | 2.7689 | 0.47748 | 0.16538 |
| Tryptophan metabolism | 79 | 2 | 2.7234 | 0.47748 | 0.14349 |
| Thiamine metabolism | 24 | 1 | 2.1002 | 0.75342 | 0 |
| Alanine, aspartate and glutamate metabolism | 24 | 1 | 2.1002 | 0.75342 | 0.17664 |
| Pantothenate and CoA biosynthesis | 27 | 1 | 1.9898 | 0.78126 | 0 |
| Propanoate metabolism | 35 | 1 | 1.7499 | 0.89156 | 0 |
| Ubiquinone and other terpenoid-quinone biosynthesis | 36 | 1 | 1.7242 | 0.89156 | 0 |
| Butanoate metabolism | 40 | 1 | 1.6286 | 0.92328 | 0 |
| Histidine metabolism | 44 | 1 | 1.5431 | 0.92357 | 0.00051 |
| Fructose and mannose metabolism | 48 | 1 | 1.4658 | 0.92357 | 0 |
| Glycine, serine and threonine metabolism | 48 | 1 | 1.4658 | 0.92357 | 0 |
| Tyrosine metabolism | 76 | 1 | 1.0737 | 1 | 0.04724 |
| Purine metabolism | 92 | 1 | 0.92066 | 1 | 0.00969 |
| Porphyrin and chlorophyll metabolism | 104 | 1 | 0.8263 | 1 | 0 |

1. **HC vs. PCOS-MS**

| **Pathway** | **Total** | **Hits** | **-ln(p)** | **FDR** | **Impact** |
| --- | --- | --- | --- | --- | --- |
| Aminoacyl-tRNA biosynthesis | 75 | 8 | 17.168 | 2.8009E-06 | 0.11268 |
| Nitrogen metabolism | 39 | 4 | 8.5595 | 0.0076684 | 0 |
| Phenylalanine metabolism | 45 | 4 | 7.9956 | 0.0089854 | 0.15056 |
| Phenylalanine, tyrosine and tryptophan biosynthesis | 27 | 3 | 6.8282 | 0.017325 | 0.008 |
| Valine, leucine and isoleucine biosynthesis | 27 | 3 | 6.8282 | 0.017325 | 0.03975 |
| Valine, leucine and isoleucine degradation | 40 | 3 | 5.676 | 0.045697 | 0.02232 |
| Tryptophan metabolism | 79 | 3 | 3.791 | 0.25799 | 0.14349 |
| Glutathione metabolism | 38 | 2 | 3.3509 | 0.35054 | 0.01095 |
| D-Arginine and D-ornithine metabolism | 8 | 1 | 2.7884 | 0.54683 | 0 |
| Biotin metabolism | 11 | 1 | 2.4811 | 0.60836 | 0 |
| D-Glutamine and D-glutamate metabolism | 11 | 1 | 2.4811 | 0.60836 | 0.1123 |
| Arginine and proline metabolism | 77 | 2 | 2.1058 | 0.81163 | 0.16538 |
| Thiamine metabolism | 24 | 1 | 1.7489 | 0.98455 | 0 |
| Alanine, aspartate and glutamate metabolism | 24 | 1 | 1.7489 | 0.98455 | 0.17664 |
| Pantothenate and CoA biosynthesis | 27 | 1 | 1.6421 | 0.98455 | 0 |
| Porphyrin and chlorophyll metabolism | 104 | 2 | 1.625 | 0.98455 | 0.01101 |
| Lysine biosynthesis | 32 | 1 | 1.4904 | 1 | 0.09993 |
| Propanoate metabolism | 35 | 1 | 1.4117 | 1 | 0 |
| Ubiquinone and other terpenoid-quinone biosynthesis | 36 | 1 | 1.3871 | 1 | 0 |
| Butanoate metabolism | 40 | 1 | 1.2962 | 1 | 0 |
| Histidine metabolism | 44 | 1 | 1.2153 | 1 | 0.00051 |
| Lysine degradation | 47 | 1 | 1.1601 | 1 | 0.14675 |
| Fructose and mannose metabolism | 48 | 1 | 1.1426 | 1 | 0 |
| Glycine, serine and threonine metabolism | 48 | 1 | 1.1426 | 1 | 0 |
| Tyrosine metabolism | 76 | 1 | 0.78156 | 1 | 0.04724 |
| Purine metabolism | 92 | 1 | 0.64539 | 1 | 0.00969 |
