# Supplementary material for: How to Screen and Prevent Metabolic Syndrome in Patients of PCOS Early: Implications From Metabolomics
Source: Front Endocrinol (Lausanne). 2021 Jun 2;12:659268. doi: 10.3389/fendo.2021.659268 (PMC8207510; doi:10.3389/fendo.2021.659268)
Supplement: Supplementary file 4 [file Table_3.docx]

**Supplement Table 3. Correlation analysis between different metabolites and clinical indicators in PCOS-MS vs. PCOS**

| **Indicators** | **compound name** | **correlation coefficient** | **P value** |
| --- | --- | --- | --- |
| HOMA-IR | carnitine C18:1 | 0.431095220568905 | 0.000105945163569484 |
| AI | LPC 20:3 | 0.42627389896485 | 0.000110862641118007 |
| TG | carnitine 4:0 | 0.425678474286128 | 0.000113609890040806 |
| FINS | lactamide | 0.425382845912531 | 0.0001149971441072 |
| apoB/apoA | leucine | 0.422937890276434 | 0.000127084762679913 |
| BMI | Glutamic acid | 0.422667402938087 | 0.000128491749607628 |
| FINS | carnitine C18:1 | 0.422188629678276 | 0.000131017482886863 |
| HDL-C | leucine | -0.415589561053671 | 0.000170833909981771 |
| SHBG | carnitine C5:0 | -0.415421724837407 | 0.000171978538995448 |
| HOMA-β | GUDCA | 0.417056627582943 | 0.000183946294294173 |
| FAI | carnitine C5:0 | 0.416267942583732 | 0.000189611036103722 |
| WHR | DL-Proline | 0.411166548269169 | 0.0002034681708905 |
| FINS | Tryptophan | 0.40820243184598 | 0.000228450177162689 |
| AI | Phenylalanine | 0.407568796796205 | 0.000234143745963736 |
| HOMA-β | leucine | 0.409642988590357 | 0.000243961978570054 |
| WC | Glutamic acid | 0.405267401644165 | 0.000255938133024074 |
| LDL-C | lactamide | 0.401590588351036 | 0.000294653829650645 |
